# LVEF-defined septic cardiomyopathy modifies the association between the prognostic nutritional index and mortality in adults with sepsis: a 72-h landmark analysis

**DOI:** 10.3389/fnut.2026.1903225

**Published:** 2026-10-06

**Authors:** Xianju Wang, Min Cheng, Fengqi Ruan, Chong Chen, Shuai Wang

**Affiliations:** 1 Department of Emergency, Linyi People's Hospital, Linyi, Shandong, China; 2 Department of Emergency Surgery, Linyi People’s Hospital, Linyi, Shandong, China; 3 Department of Critical Care Medicine, Linyi People's Hospital, Linyi, Shandong, China

**Keywords:** effect modification, immunonutrition, left ventricular ejection fraction, mortality, prognostic nutritional index, sepsis, septic cardiomyopathy

## Abstract

**Background:**

The prognostic nutritional index (PNI) combines albumin and lymphocyte count and may capture nutrition-related immune-inflammatory status in sepsis. Whether its association with mortality differs by left ventricular ejection fraction (LVEF)-defined septic cardiomyopathy (SCM) is unclear.

**Methods:**

We conducted a retrospective 72-h landmark study of MIMIC-IV adults with Sepsis-3 who survived to the landmark, had clinically indicated pre-landmark echocardiographic LVEF assessment, and had calculable early PNI. The prespecified primary analysis tested the lower-PNI-by-LVEF-defined-SCM interaction for 28-day mortality in an adjusted Cox model. Ninety-day and sensitivity analyses were supportive or exploratory. A single-center cohort was examined separately, not as formal validation.

**Results:**

Among 1,976 patients, 240 had LVEF-defined SCM; 506 and 633 died by 28 and 90 days, respectively. Each 5-point lower PNI was associated with higher 28-day mortality without SCM (adjusted hazard ratio [HR], 1.18; 95% CI, 1.11–1.24) but not with LVEF-defined SCM (HR, 0.96; 95% CI, 0.87–1.05); the ratio of HRs was 0.82 (95% CI, 0.73–0.91; *p* < 0.001). The secondary 90-day ratio was 0.84 (95% CI, 0.76–0.94; nominal *p* = 0.001). Absolute-risk and restricted-mean-survival-time analyses supported attenuation, whereas same-calendar-day PNI pairing and some alternative LVEF definitions attenuated the interaction estimates toward the null. The external estimate was imprecise and statistically inconclusive (ratio of odds ratios, 0.89; 95% CI, 0.51–1.55).

**Conclusion:**

Among selected 72-h survivors with clinically indicated pre-landmark echocardiography, the association between lower PNI and 28-day mortality was attenuated with LVEF-defined SCM. PNI should not be used alone for nutritional or clinical decision-making.

## Introduction

Sepsis is life-threatening organ dysfunction caused by a dysregulated host response to infection (1). In critically ill patients, inflammation, altered substrate use, and organ failure make nutritional status difficult to interpret, even though it remains clinically important (2, 3). Myocardial dysfunction adds another layer of prognostic uncertainty. Reversible myocardial depression in septic shock was recognized decades ago (4). Septic cardiomyopathy (SCM) is now understood as a heterogeneous syndrome that may involve left ventricular systolic or diastolic dysfunction, right ventricular impairment, strain abnormalities, and load-dependent changes in contractility (5, 6). This raises a practical question: does a nutrition-related immune-inflammatory marker carry the same prognostic information once early myocardial dysfunction is present?

The prognostic nutritional index (PNI), calculated from serum albumin and peripheral blood lymphocyte count, was introduced as a simple composite index (7). During acute sepsis, neither component reflects nutritional status alone. Albumin is shaped by systemic inflammation, capillary leakage, acute illness severity, hepatic synthesis, fluid balance and dilution, and nutritional reserve (8). Lymphopenia is common in sepsis and closely tied to sepsis-associated immune dysfunction (9). PNI is therefore better interpreted as a nutrition-related immune-inflammatory composite marker than as a direct measure of nutritional status. Two retrospective sepsis cohorts reported associations between lower PNI and higher mortality (10, 11). A systematic review and meta-analysis also reported an inverse association between PNI and mortality in sepsis (12). Whether this association differs by early cardiac phenotype remains uncertain.

SCM may alter this prognostic setting. In patients without overt myocardial dysfunction, a low PNI may track mortality through the expected inflammatory, immune, and nutritional pathway. In patients with SCM, early outcomes may be driven more strongly by cardiac performance, hemodynamic instability, and the need for cardiovascular support. That could weaken the prognostic signal carried by baseline PNI. This is also a different question from whether PNI is associated with the occurrence of SCM (13). Here, the question is whether LVEF-defined SCM modifies the association between early PNI and subsequent mortality.

We examined whether LVEF-defined SCM modifies the association between early PNI and mortality in adults with sepsis. Using MIMIC-IV (14), we applied a 72-h landmark design among patients who survived to the landmark, had clinically indicated pre-landmark TTE-LVEF assessment, and had calculable early PNI. This ensured that early PNI and LVEF-defined SCM status were defined before mortality follow-up began. We also examined the interaction estimate and its precision in a single-center external exploratory cohort without treating that analysis as formal validation. We hypothesized that the inverse association between PNI and mortality would be more evident in patients without LVEF-defined SCM and attenuated in those with this phenotype.

## Methods

### Study design and data sources

We conducted a retrospective 72-h landmark cohort study to evaluate whether the association between early PNI and mortality differed according to LVEF-defined SCM status. The primary analysis used MIMIC-IV v3.1 ICU data (14) linked to a locally derived table of structured LVEF measurements extracted from clinical TTE reports. Exposure and covariate windows were anchored to ICU admission, and survival follow-up began at the 72-h landmark. The 72-h landmark aligned follow-up with completion of the early PNI (through 48 h) and TTE-LVEF (through 72 h) assessment windows. This temporal ordering is consistent with landmark-analysis principles (15) and reduces immortal-time bias (16).

The external exploratory cohort comprised 272 consecutive adults with sepsis treated in multiple adult ICUs at Linyi People’s Hospital from January 2023 through December 2025. Specific ICU subtype was not retained in a standardized field. Sepsis was identified from clinician-documented diagnoses rather than by reapplying the MIMIC-IV database algorithm. This cohort was used only to assess the interaction estimate outside MIMIC-IV and not as a formal external validation. MIMIC-IV cohort construction is shown in Figure 1.

**Figure 1:**
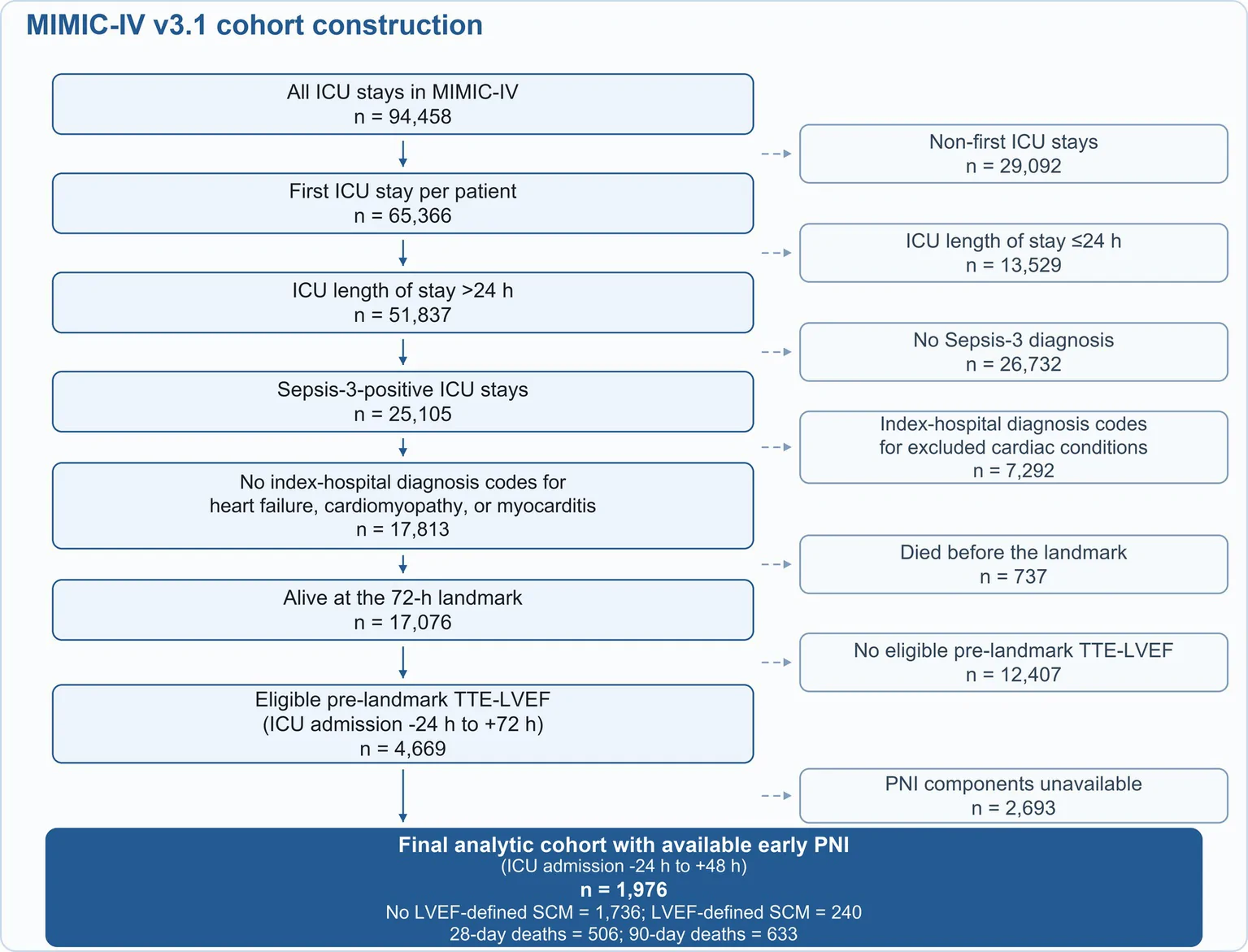
Construction of the MIMIC-IV v3.1 72-h landmark analytic cohort. ICU admission was the index time and the landmark was 72 h later; survival to the landmark did not require continued ICU or hospital stay. Sepsis-3 required suspected infection plus a SOFA score ≥2. PNI was calculated as albumin (g/L) + 5 × lymphocyte count (10^9^/L). LVEF-defined SCM was any eligible LVEF <50%, or a >10-percentage-point decline from an earlier eligible value with the later LVEF <55%. Cardiac diagnosis-code exclusions are described in Methods. ICU, intensive care unit; LVEF, left ventricular ejection fraction; MIMIC-IV, Medical Information Mart for Intensive Care IV; PNI, prognostic nutritional index; SCM, septic cardiomyopathy; SOFA, Sequential Organ Failure Assessment; TTE, transthoracic echocardiography.

### Study cohorts and eligibility criteria

We used the Sepsis-3 framework (1) implemented in mimiciv_derived.sepsis3. Adults aged 18 years or older were identified by selecting sepsis3 = TRUE records during the first ICU stay lasting more than 24 h. Suspected infection required antibiotic administration within 72 h after microbiologic culture or culture collection within 24 h after antibiotic initiation. Organ dysfunction required a rolling 24-h SOFA score of at least 2 from 48 h before through 24 h after suspected infection, assuming a baseline SOFA score of 0. The earliest qualifying Sepsis-3 event in each ICU stay was retained.

Before landmark-cohort construction, we excluded patients with diagnosis codes for heart failure, cardiomyopathy including ischemic cardiomyopathy, or myocarditis in the index hospitalization record. Eligible patients had to be alive at 72 h and have clinically indicated, rather than protocol-mandated, pre-landmark TTE-LVEF assessment. They also required albumin and lymphocyte measurements sufficient to calculate early PNI. Survival to the landmark did not require continued ICU or hospital stay. The resulting MIMIC-IV cohort should be interpreted as a cardiovascularly evaluated subset of 72-h sepsis survivors.

### Clinical data and exposure definitions

Prespecified variables included demographics, admission characteristics, comorbidities, SOFA and APS III scores, laboratory measurements, TTE-derived LVEF, organ support, and mortality outcomes. Invasive mechanical ventilation and vasopressor use were defined during the first 24 h after ICU admission. Dobutamine or milrinone use before the landmark was examined in sensitivity analyses. Coronary artery disease was identified from the Charlson myocardial-infarction indicator or diagnosis codes recorded during the index hospitalization.

Anatomical infection source and a validated Sepsis-3 septic-shock indicator were not available in the frozen analytic dataset. Detailed vasopressor dose, norepinephrine-equivalent exposure, temporally aligned cumulative fluid balance, and glucocorticoid exposure were also unavailable and were not modeled.

SCM was operationalized as LVEF-defined left ventricular systolic dysfunction rather than the complete clinical spectrum of SCM. In MIMIC-IV, eligible TTE-LVEF measurements extended from 24 h before ICU admission to the 72-h landmark and were bounded by the hospitalization interval. SCM was present when any eligible LVEF was below 50%, or when LVEF fell by more than 10 percentage points from an earlier eligible value and the later value was below 55%. All eligible measurements contributed to classification, and the lowest LVEF was used for descriptive summaries. In the external cohort, clinically obtained TTE-LVEF was required from 6 h before through 24 h after ICU admission. Because serial measurements were not consistently available, SCM was defined by any available LVEF below 50% in that window.

LVEF sensitivity analyses removed the decline criterion and applied thresholds below 50, 45%, or 40% to either any eligible measurement or the first eligible TTE. First and lowest LVEF were also categorized as at least 50, 40–49%, or below 40% for joint interaction tests. Ascertainment audits summarized eligible TTE counts, timing relative to ICU admission and vasoactive therapy, recorded LVEF source, and numeric heaping.

Early PNI was calculated as albumin (g/L) + 5 × peripheral blood lymphocyte count (10^9^/L). Albumin and lymphocyte measurements were independently selected as the earliest valid values from 24 h before through 48 h after ICU admission and were not required to occur on the same day. Timing sensitivity analyses reconstructed PNI from the closest pair on the same calendar day and, separately, from pairs no more than 24 h apart. In regression models, lower PNI was scaled per 5-point decrease as PNI_low5 = −PNI/5 and mean-centered before interaction modeling.

### Outcomes

The single primary outcome was 28-day all-cause mortality after the 72-h landmark. Ninety-day all-cause mortality was a secondary supportive outcome. We used mimiciv_hosp.admissions.deathtime when available and otherwise used the patient-level death date in mimiciv_hosp.patients.dod. Recorded deaths after the landmark and within each horizon were counted as events, including deaths recorded after hospital discharge. Patients without a recorded death were administratively censored at 28 or 90 days. The external exploratory endpoint was chart-verified in-hospital death; outcome data were complete.

### Missing data

Values outside prespecified physiologic ranges were set to missing before analysis, and candidate covariates with more than 30% missingness were excluded from multivariable models. After range checks in MIMIC-IV, missingness was 13.77% for body mass index, 12.10% for lactate, and 0.25% for white blood cell count. In the external cohort, missingness was 25.37% for body mass index, 6.62% for SOFA score, 2.57% for lactate, and 2.21% for blood urea nitrogen. Other retained analytic variables were complete (Supplementary Table S2, Panel D1).

Missing covariates were imputed by chained equations with 10 datasets, 20 iterations, predictive mean matching, and random seed 2025. In MIMIC-IV, the predictor matrix included age, sex, body mass index, lactate, white blood cell count, SOFA, APS III, SCM, LVEF, albumin, lymphocyte count, creatinine, platelet count, blood urea nitrogen, mechanical ventilation, vasopressor use, coronary artery disease, diabetes, hypertension, chronic kidney disease, chronic obstructive pulmonary disease, and complete mortality and follow-up variables. Patient identifiers and PNI were not predictors. Complete outcomes and follow-up times were included as auxiliary predictors but were not imputed. Albumin, lymphocyte count, derived PNI, LVEF-defined SCM, outcomes, and follow-up times were not imputed. Corresponding observed variables were used in the external-cohort imputation model. As an interaction-compatibility sensitivity analysis in MIMIC-IV, a centered PNI-by-SCM product was added as a complete predictor of BMI, lactate, and white blood cell count; deterministic PNI itself remained excluded because its complete albumin and lymphocyte components were already included. Trace plots, observed-versus-imputed distributions, residual missingness, and logged events were examined. Extending the original chains from 5 to 20 iterations did not change the reported estimates.

### Statistical analysis

Because continuous-variable distributions were skewed, they were summarized as medians with interquartile ranges and compared using Mann–Whitney *U* tests. Categorical variables were summarized as counts and percentages and compared using chi-square or Fisher exact tests. Absolute standardized mean differences described within-cohort, between-cohort, and selection-related imbalances; values above 0.10 were considered meaningful.

The prespecified primary interaction test was the lower-PNI-by-LVEF-defined-SCM term in the fully adjusted 28-day Cox model. The 90-day model was secondary. Model 1 was unadjusted, and Model 2 adjusted for age, sex, and body mass index. Model 3 additionally adjusted for SOFA score, log-transformed lactate and creatinine, coronary artery disease, diabetes, hypertension, chronic kidney disease, chronic obstructive pulmonary disease, invasive mechanical ventilation, and vasopressor use. These variables were treated as prespecified adjustment variables. Mechanical ventilation and vasopressor use could also represent disease-severity markers or potential mediators, so models excluding both were examined.

Each Cox model was fitted separately in the 10 imputed datasets. Regression coefficients and variances were combined using Rubin’s rules, and two-sided interaction *p* values used Wald *t* tests with Barnard-Rubin degrees of freedom. Stratum-specific PNI HRs were obtained as linear combinations of the PNI and interaction coefficients, and the ratio of HRs compared the LVEF-defined SCM stratum with the No SCM stratum. Model 3 estimated 16 regression parameters. Model complexity was summarized using events per parameter for the full cohort and, conservatively, the SCM stratum. Main and alternative model specifications are reported in Supplementary Table S1, Panel A.

### Selection-bias analysis

The selection target population comprised all 17,076 patients alive at the landmark. We first compared patients with and without eligible pre-landmark TTE-LVEF, then compared the final analytic cohort with all patients not included. Comparisons used unweighted baseline characteristics, absolute standardized mean differences, and crude mortality outcomes. Crude outcome differences described the selection process and were not interpreted as effects of receiving TTE.

Stabilized average-treatment-effect inverse-probability-of-selection weights were estimated from prespecified logistic selection models. Predictors covered demographics, admission type and source, illness severity, laboratory measures, comorbidities, organ support, and missingness indicators. One model targeted eligible TTE-LVEF, and a stricter model targeted entry into the final TTE-LVEF and PNI-complete cohort. Balance was adequate when every weighted absolute standardized mean difference was below 0.10. Selection-weighted Cox models used robust sandwich variances within each imputed dataset and Rubin-rule pooling. Trimming weights at the 1st and 99th percentiles was examined (Supplementary Table S2, Panel C).

### Sensitivity and exploratory analyses

Model-specification sensitivity analyses used a demographics-and-comorbidities model, a fully adjusted model excluding organ support, and models adding APS III and pre-landmark inotrope use. We also examined alternative LVEF definitions, PNI timing rules, and PNI winsorization at the 1st and 99th percentiles. Exploratory E-values used a common-outcome approximation from HRs to risk ratios. Because the interaction estimate is a ratio of HRs, its E-value was interpreted only as an HR-scale sensitivity indicator.

The proportional-hazards assumption was assessed using Schoenfeld residuals in each completed dataset; ranges are reported because these tests lack a standard Rubin-pooling procedure. Time-varying Cox models used *g*(*t*) = log(1 + *t*) − log(29) for lower PNI, its interaction with SCM, and covariates showing PH deviations. Pointwise interaction estimates were obtained at prespecified days, while the PNI-by-SCM-by-*g*(*t*) coefficient provided the formal trend test. Window-specific Cox models examined days 0–28 and days 28–90 among 28-day survivors. Follow-up was complete to each horizon without earlier censoring, so restricted survival time was directly observed. Adjusted RMST analyses at 28 and 90 days used identity-link linear regression with HC0 robust covariance. All non-PH models used Model 3 variables and Rubin-rule pooling (Supplementary Table S1, Panel C; Supplementary Figure S2).

Multicollinearity was assessed using variance inflation factors, and model-term correlations were summarized using Spearman coefficients (Supplementary Table S2, Panel A; Supplementary Figure S1). Natural cubic spline models used 3 degrees of freedom and default quantile-based knots from splines::ns, with PNI = 35 as the reference. Pointwise log-HR contrasts were pooled using Rubin’s rules. Nonlinearity and spline-interaction terms were tested using Rubin D1 joint tests. Spline analyses used original and winsorized PNI (Supplementary Table S1, Panels A3,B).

Separate fully adjusted fixed-time logistic models compared PNI 30 with PNI 40 within SCM strata. Risks were marginally standardized over the analytic-cohort covariate distribution. Risk differences and percentile 95% CIs used 1,000 nonparametric bootstrap resamples with seed 2025. Exploratory component models evaluated albumin and lymphocyte count separately and jointly. SCM-stratified C-index estimates were pooled on the logit scale across imputations (Supplementary Table S1, Panels B,D).

In the external cohort, Firth penalized logistic regression modeled in-hospital death. The limited clinical model included age, sex, body mass index, SOFA score, and log-transformed lactate and creatinine. Models were fitted in each imputed dataset and combined using Rubin’s rules. Interaction *p* values used two-sided Wald *t* tests with Barnard-Rubin degrees of freedom (Supplementary Table S2, Panel B). Only the primary 28-day interaction test was treated as confirmatory. No multiplicity adjustment was applied to that single test; all secondary, supportive, and exploratory *p* values were nominal. Analyses used R version 4.5.3 (R Foundation for Statistical Computing, Vienna, Austria).

## Results

### Study population and baseline characteristics

Among 17,076 patients alive at the 72-h landmark, 4,669 had eligible pre-landmark TTE-LVEF data. Of these, 1,976 also had calculable early PNI and formed the final analytic cohort; 12,407 landmark survivors lacked eligible TTE-LVEF, and 2,693 patients with eligible TTE-LVEF lacked complete PNI components (Figure 1). The final cohort included 1,736 patients without SCM and 240 with LVEF-defined SCM. There were 506 deaths by 28 days and 633 by 90 days after the landmark.

Median PNI was 35.3 (IQR, 29.9–41.3) without SCM and 36.6 (IQR, 28.2–43.5) with LVEF-defined SCM (*p* = 0.330). Albumin, lymphocyte count, and SOFA score were also similar between strata. Patients with LVEF-defined SCM had lower LVEF, higher lactate and white blood cell counts, more coronary artery disease, and more frequent invasive mechanical ventilation and vasopressor use. Unadjusted 28-day mortality was 24.5% without SCM and 33.3% with SCM; corresponding 90-day mortality was 30.9 and 40.0% (Table 1). These outcome comparisons were descriptive.

**Table 1:** Baseline characteristics of the MIMIC-IV 72-h landmark cohort and the external exploratory cohort, stratified by LVEF-defined SCM.

Variable	MIMIC-IV cohort			*p* value	External cohort			*p* value
	Overall (*n* = 1,976)	No SCM (*n* = 1,736)	SCM (*n* = 240)		Overall (*n* = 272)	No SCM (*n* = 199)	SCM (*n* = 73)	
Demographics and severity								
Age, years	62.0 [50.0, 73.0]	62.0 [50.0, 73.0]	62.0 [52.0, 72.5]	0.717	69.0 [56.0, 77.5]	69.0 [55.0, 77.0]	71.0 [57.0, 78.0]	0.541
Female sex, *n* (%)	889 (45.0)	804 (46.3)	85 (35.4)	0.001	101 (37.1)	72 (36.2)	29 (39.7)	0.592
BMI, kg/m^2^	28.1 [24.1, 33.2]	28.2 [24.3, 33.5]	26.6 [23.1, 31.2]	<0.001	23.6 [21.0, 26.7]	23.5 [21.0, 27.1]	23.8 [20.6, 25.0]	0.320
SOFA score	4.0 [2.0, 5.0]	4.0 [2.0, 5.0]	4.0 [2.0, 5.0]	0.760	5.0 [3.0, 8.0]	5.0 [3.0, 7.0]	6.0 [3.0, 8.0]	0.945
Cardiac and nutritional markers								
LVEF, %	65.0 [57.0, 77.5]	68.0 [60.0, 77.5]	40.0 [30.0, 45.0]	<0.001	55.5 [48.5, 58.0]	58.0 [55.0, 58.0]	40.0 [36.0, 46.0]	<0.001
Albumin, g/L	30.0 [25.0, 35.0]	30.0 [25.0, 35.0]	31.0 [24.0, 36.0]	0.569	30.9 [27.9, 34.4]	31.1 [28.2, 34.3]	29.9 [26.4, 35.1]	0.269
Lymphocyte, ×10^9^/L	0.9 [0.5, 1.5]	0.9 [0.5, 1.5]	0.9 [0.5, 1.5]	0.748	0.7 [0.4, 1.1]	0.6 [0.4, 1.0]	0.7 [0.5, 1.2]	0.195
PNI	35.4 [29.7, 41.6]	35.3 [29.9, 41.3]	36.6 [28.2, 43.5]	0.330	34.8 [31.1, 38.7]	35.0 [31.2, 38.5]	34.1 [30.8, 39.3]	0.615
Laboratory markers								
Lactate, mmol/L	2.3 [1.5, 4.4]	2.3 [1.5, 4.0]	3.5 [1.9, 6.5]	<0.001	1.8 [1.2, 2.9]	1.5 [1.1, 2.6]	2.5 [1.8, 3.4]	<0.001
Creatinine, μmol/L	115 [80, 203]	115 [80, 203]	124 [88, 203]	0.113	132 [76, 270]	130 [72, 267]	147 [91, 289]	0.247
WBC, ×10^9^/L	14.8 [9.9, 21.0]	14.4 [9.7, 20.5]	17.0 [11.6, 23.2]	<0.001	12.4 [8.1, 17.5]	12.5 [8.0, 18.0]	11.5 [8.1, 16.8]	0.342
Platelet, ×10^9^/L	146.0 [85.5, 214.5]	143.0 [84.0, 212.0]	156.5 [96.5, 226.5]	0.073	172.0 [93.0, 255.5]	178.0 [100.0, 266.0]	152.0 [89.0, 221.0]	0.203
BUN, mmol/L	9.3 [6.1, 15.7]	9.3 [6.1, 16.1]	9.3 [6.4, 14.5]	0.572	12.7 [7.8, 21.1]	12.0 [7.4, 20.6]	14.8 [10.6, 24.0]	0.118
Comorbidities								
Coronary artery disease, *n* (%)	578 (29.3)	447 (25.7)	131 (54.6)	<0.001	35 (12.9)	23 (11.6)	12 (16.4)	0.287
Diabetes mellitus, *n* (%)	501 (25.4)	436 (25.1)	65 (27.1)	0.511	81 (29.8)	55 (27.6)	26 (35.6)	0.202
Hypertension, *n* (%)	1,107 (56.0)	970 (55.9)	137 (57.1)	0.724	105 (38.6)	86 (43.2)	19 (26.0)	0.010
Chronic kidney disease, *n* (%)	329 (16.6)	281 (16.2)	48 (20.0)	0.137	23 (8.5)	15 (7.5)	8 (11.0)	0.369
COPD, *n* (%)	449 (22.7)	389 (22.4)	60 (25.0)	0.369	21 (7.7)	13 (6.5)	8 (11.0)	0.226
Organ support								
Mechanical ventilation, *n* (%)	1,145 (57.9)	981 (56.5)	164 (68.3)	<0.001	153 (56.2)	125 (62.8)	28 (38.4)	<0.001
Vasopressor use, *n* (%)	913 (46.2)	776 (44.7)	137 (57.1)	<0.001	145 (53.3)	121 (60.8)	24 (32.9)	<0.001
Outcomes; descriptive only								
28-day mortality, *n* (%)	506 (25.6)	426 (24.5)	80 (33.3)	–	NA	NA	NA	–
90-day mortality, *n* (%)	633 (32.0)	537 (30.9)	96 (40.0)	–	NA	NA	NA	–
In-hospital death, *n* (%)	NA	NA	NA	–	77 (28.3)	64 (32.2)	13 (17.8)	–

BMI, body mass index; BUN, blood urea nitrogen; COPD, chronic obstructive pulmonary disease; LVEF, left ventricular ejection fraction; MIMIC-IV, Medical Information Mart for Intensive Care IV; PNI, prognostic nutritional index; SCM, septic cardiomyopathy; SOFA, Sequential Organ Failure Assessment; WBC, white blood cell count. Data are median [Q1, Q3] or *n* (%), based on observed pre-imputation values; missingness and imputation are detailed in Supplementary Table S2, Panel D1. Within-cohort absolute standardized mean differences are reported in Supplementary Table S3. *p* values used Mann–Whitney *U*, chi-square, or Fisher’s exact tests, as appropriate. NA, not available. Outcomes were descriptive and not formally compared (−).

The external exploratory cohort included 272 patients, of whom 199 had no SCM and 73 had LVEF-defined SCM. Median PNI was 35.0 (IQR, 31.2–38.5) and 34.1 (IQR, 30.8–39.3), respectively (*p* = 0.615). Seventy-seven patients died in hospital (Table 1).

### Between-cohort heterogeneity

The MIMIC-IV and external cohorts differed substantially in case mix. The external cohort was older and had lower body mass index, higher SOFA scores, a higher prevalence of LVEF-defined SCM, lower LVEF and lymphocyte counts, higher blood urea nitrogen, and more frequent vasopressor use. Overall median PNI was similar between cohorts (35.4 [IQR, 29.7–41.6] versus 34.8 [IQR, 31.1–38.7]; *p* = 0.373), although the absolute standardized mean difference was 0.112. These differences supported treating the external cohort as exploratory rather than as a formal validation cohort (Table 2).

**Table 2:** Between-cohort heterogeneity in baseline characteristics.

Variable	MIMIC-IV cohort (*n* = 1,976)	External cohort (*n* = 272)	*p* value	Absolute SMD
Demographics and severity				
Age, years	62.0 [50.0, 73.0]	69.0 [56.0, 77.5]	<0.001	0.331
Female sex, *n* (%)	889 (45.0)	101 (37.1)	0.014	0.160
BMI, kg/m^2^	28.1 [24.1, 33.2]	23.6 [21.0, 26.7]	<0.001	0.730
SOFA score	4.0 [2.0, 5.0]	5.0 [3.0, 8.0]	<0.001	0.590
SCM prevalence, *n* (%)	240 (12.1)	73 (26.8)	<0.001	0.377
Cardiac and nutritional markers				
LVEF, %	65.0 [57.0, 77.5]	55.5 [48.5, 58.0]	<0.001	0.876
Albumin, g/L	30.0 [25.0, 35.0]	30.9 [27.9, 34.4]	0.021	0.122
Lymphocyte count, ×10^9^/L	0.9 [0.5, 1.5]	0.7 [0.4, 1.1]	<0.001	0.313
PNI	35.4 [29.7, 41.6]	34.8 [31.1, 38.7]	0.373	0.112
Laboratory markers				
Lactate, mmol/L	2.3 [1.5, 4.4]	1.8 [1.2, 2.9]	<0.001	0.351
Creatinine, μmol/L	115 [80, 203]	132 [76, 270]	0.024	0.242
WBC, ×10^9^/L	14.8 [9.9, 21.0]	12.4 [8.1, 17.5]	<0.001	0.282
Platelet count, ×10^9^/L	146.0 [85.5, 214.5]	172.0 [93.0, 255.5]	0.001	0.198
BUN, mmol/L	9.3 [6.1, 15.7]	12.7 [7.8, 21.1]	<0.001	0.370
Comorbidities				
Coronary artery disease, *n* (%)	578 (29.3)	35 (12.9)	<0.001	0.410
Diabetes mellitus, *n* (%)	501 (25.4)	81 (29.8)	0.118	0.099
Hypertension, *n* (%)	1,107 (56.0)	105 (38.6)	<0.001	0.354
Chronic kidney disease, *n* (%)	329 (16.6)	23 (8.5)	<0.001	0.249
COPD, *n* (%)	449 (22.7)	21 (7.7)	<0.001	0.427
Organ support				
Mechanical ventilation, *n* (%)	1,145 (57.9)	153 (56.2)	0.596	0.034
Vasopressor use, *n* (%)	913 (46.2)	145 (53.3)	0.028	0.142

Data are presented as median [Q1, Q3] or *n* (%). Baseline summaries were calculated from observed data before imputation. *p* values were calculated using the Mann–Whitney *U* test, chi-square test, or Fisher’s exact test, as appropriate. Absolute SMDs were calculated using individual-level data; for continuous variables, SMDs used means and pooled standard deviations. Values >0.10 were considered indicative of meaningful between-cohort imbalance.

BUN, blood urea nitrogen; COPD, chronic obstructive pulmonary disease; LVEF, left ventricular ejection fraction; MIMIC-IV, Medical Information Mart for Intensive Care IV; PNI, prognostic nutritional index; SCM, septic cardiomyopathy; SMD, standardized mean difference; SOFA, Sequential Organ Failure Assessment; WBC, white blood cell.

### Primary and secondary interaction analyses

In the prespecified primary analysis, the association between lower PNI and 28-day mortality differed according to LVEF-defined SCM status (Figure 2; Supplementary Table S1, Panel A1). Each 5-point lower PNI was associated with higher mortality among patients without SCM (adjusted HR, 1.18; 95% CI, 1.11–1.24; *p* < 0.001), whereas no corresponding association was detected among patients with LVEF-defined SCM (HR, 0.96; 95% CI, 0.87–1.05; *p* = 0.384). The ratio of HRs was 0.82 (95% CI, 0.73–0.91; Rubin-pooled Wald *p* < 0.001).

**Figure 2:**
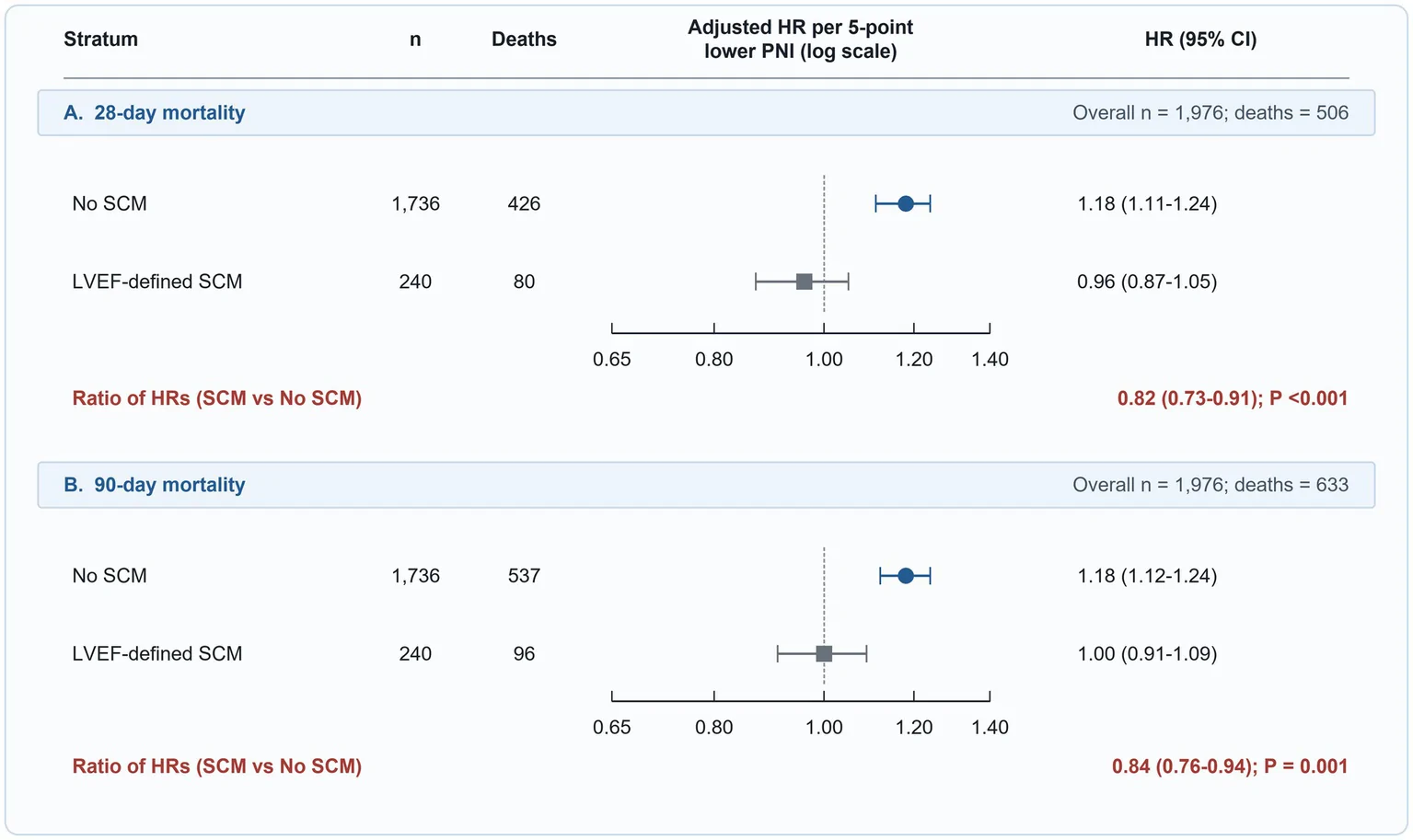
Adjusted associations of lower PNI with 28- and 90-day all-cause mortality according to LVEF-defined SCM in MIMIC-IV. HRs are per 5-point lower PNI, adjusted for age, sex, body mass index, SOFA score, log-transformed lactate and creatinine, coronary artery disease, diabetes, hypertension, chronic kidney disease, chronic obstructive pulmonary disease, invasive mechanical ventilation, and vasopressor use. The ratio of HRs (SCM versus No SCM) quantifies effect modification; values <1 indicate attenuation with SCM. The 28-day interaction was the prespecified primary test; the 90-day analysis was secondary and supportive. Ratios of HRs were calculated from unrounded model coefficients. Estimates from 10 imputed datasets were combined using Rubin’s rules; interaction *p* values were obtained from two-sided Wald *t* tests with Barnard–Rubin degrees of freedom. Because the proportional hazards assumption was not fully satisfied, HRs represent average associations over each horizon; proportional-hazards diagnostics, time-varying Cox models, and restricted mean survival time analyses are reported in Supplementary Figure S2 and Supplementary Tables S1 (Panel C), S2 (Panel A1). CI, confidence interval; HR, hazard ratio; LVEF, left ventricular ejection fraction; MIMIC-IV, Medical Information Mart for Intensive Care IV; PNI, prognostic nutritional index; SCM, septic cardiomyopathy; SOFA, Sequential Organ Failure Assessment.

The secondary 90-day analysis likewise showed a weaker PNI-mortality association in patients with LVEF-defined SCM. Each 5-point lower PNI was associated with higher mortality without SCM (adjusted HR, 1.18; 95% CI, 1.12–1.24; *p* < 0.001) but not with LVEF-defined SCM (HR, 1.00; 95% CI, 0.91–1.09; *p* = 0.922). The ratio of HRs was 0.84 (95% CI, 0.76–0.94; nominal *p* = 0.001). Results were similar in Models 1 and 2.

### Absolute-risk translation and nonlinear patterns

Absolute-risk analyses supported the same contrast (Figure 3; Supplementary Table S1, Panels B2,B3). Comparing PNI 30 with PNI 40, adjusted 28-day risk differences were +6.6 percentage points (95% CI, 4.1 to 9.3) without SCM and −1.0 percentage points (−5.2 to 4.6) with LVEF-defined SCM; the between-stratum contrast (SCM minus No SCM) was −7.6 percentage points (−12.9 to −1.8). Corresponding 90-day risk differences were +7.9 percentage points (4.9 to 11.1) and +1.0 percentage points (−3.4 to 7.0), with a contrast (SCM minus No SCM) of −6.9 percentage points (−12.5 to −0.3). The upper confidence bound for the 90-day contrast was close to the null.

**Figure 3:**
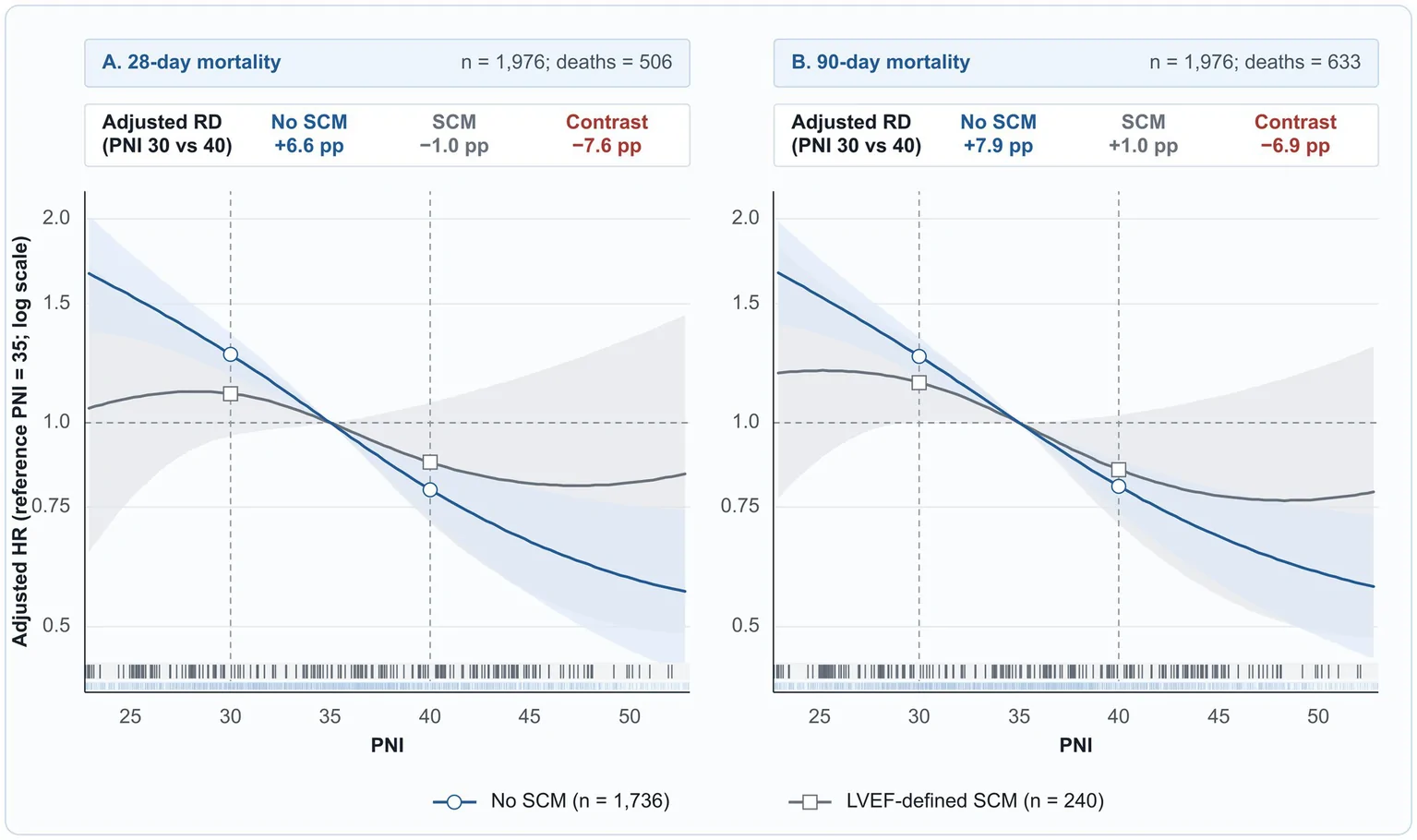
Exploratory associations of PNI with mortality according to LVEF-defined SCM status. Panels A and B show fully adjusted, Rubin-pooled Cox spline estimates using original (non-winsorized) PNI for 28-day and 90-day mortality, respectively, with PNI = 35 as the reference. Shaded areas indicate 95% CIs, and the upper dark gray and lower light blue rug marks show PNI distributions in the LVEF-defined SCM and No SCM strata, respectively. The displayed range approximates the 5th-95th percentiles of PNI in the overall cohort and is separate from the 1st-99th percentile winsorization sensitivity analysis. The No SCM and LVEF-defined SCM strata included 1,736 and 240 patients, respectively; corresponding deaths were 426 and 80 at 28 days, and 537 and 96 at 90 days. Top annotations report standardized adjusted RDs for PNI 30 versus 40 from separate fully adjusted fixed-time logistic models with 1,000 bootstrap resamples. At 28 days, RDs were +6.6 percentage points (95% CI, 4.1 to 9.3) for No SCM and −1.0 percentage points (−5.2 to 4.6) for SCM. The RD contrast (SCM minus No SCM) was −7.6 percentage points (−12.9 to −1.8). At 90 days, RDs were +7.9 percentage points (4.9 to 11.1) and +1.0 percentage points (−3.4 to 7.0), respectively. The corresponding contrast was −6.9 percentage points (−12.5 to −0.3). Estimates at sparse PNI extremes, particularly higher PNI values among patients with LVEF-defined SCM, should be interpreted cautiously. After winsorization, evidence for nonlinearity was attenuated and no longer significant, whereas the linear PNI-by-SCM interaction remained significant (Supplementary Table S1, Panels A3,B). These spline and absolute-risk analyses were supportive and exploratory rather than confirmatory. CI, confidence interval; HR, hazard ratio; LVEF, left ventricular ejection fraction; PNI, prognostic nutritional index; RD, risk difference; SCM, septic cardiomyopathy.

Natural cubic splines showed a steeper PNI-mortality gradient without SCM and a weaker gradient with LVEF-defined SCM (Figure 3; Supplementary Table S1, Panel B1). After 1st-99th percentile winsorization, evidence for nonlinearity was attenuated and no longer significant, whereas the linear interaction remained for 28-day and 90-day mortality (*p* < 0.001 and *p* = 0.005, respectively; Supplementary Table S1, Panel A3). Spline and absolute-risk analyses were supportive and exploratory.

### Non-proportional hazards and RMST analyses

Schoenfeld-residual diagnostics indicated that the proportional-hazards assumption was not fully satisfied, including for the PNI-by-SCM interaction (Supplementary Table S2, Panel A1). Fixed Cox HRs were retained as model-based summaries over each prespecified horizon and interpreted alongside the time-varying and RMST analyses. In time-varying models, the interaction HR changed from 0.81 at day 1 to 0.92 at day 28 in the 28-day analysis and from 0.80 at day 1 to 1.01 at day 90 in the 90-day analysis. Formal log-time interaction trends were not significant (*p* = 0.364 and *p* = 0.126, respectively; Supplementary Figure S2; Supplementary Table S1, Panel C3).

Window-specific estimates were 0.82 (95% CI, 0.73–0.91; *p* < 0.001) during days 0–28 and 1.08 (95% CI, 0.81–1.45; *p* = 0.597) during days 28–90 among 28-day survivors, but proportional-hazards deviations remained and the latter analysis was conditional on 28-day survival (Supplementary Table S1, Panel C4). In PH-independent analyses, each 5-point lower PNI was associated with a 0.51-day decrease in 28-day RMST (95% CI, 0.28 to 0.73 days shorter) without SCM and a 0.34-day increase (−0.22 to 0.90) with LVEF-defined SCM; the between-stratum difference (SCM minus No SCM) was +0.85 days (0.25 to 1.45; *p* = 0.005). The corresponding 90-day difference (SCM minus No SCM) was +2.51 days (0.31 to 4.71; *p* = 0.025; Supplementary Table S1, Panel C5). These results supported attenuation without establishing a significant temporal trend.

### Selection and measurement sensitivity analyses

Patients with eligible TTE-LVEF differed from those without it, and the final analytic cohort differed from the other landmark survivors (Supplementary Table S2, Panels C2–C4). For final-cohort selection, the largest absolute standardized mean difference was 0.580 before weighting and 0.081 after stabilized selection weighting. All weighted covariate terms were below 0.10. TTE-selection-weighted interaction HRs were 0.79 (95% CI, 0.69–0.90; *p* < 0.001) at 28 days and 0.83 (95% CI, 0.72–0.95; *p* = 0.006) at 90 days. With the stricter final-cohort selection model, interaction HRs were 0.78 (95% CI, 0.66–0.93; *p* = 0.005) and 0.81 (95% CI, 0.69–0.96; *p* = 0.014), and estimates remained similar after weight trimming (Supplementary Table S2, Panel C5).

Only 3 of 240 patients with primary LVEF-defined SCM met the decline criterion alone, and none died by 28 or 90 days. Removing that criterion left the interaction estimates essentially unchanged. Using LVEF below 50% on the first eligible TTE attenuated the interaction to 0.88 (95% CI, 0.78–0.99; *p* = 0.027) at 28 days and 0.91 (95% CI, 0.81–1.01; *p* = 0.075) at 90 days. At thresholds below 45 and 40%, the definition-positive groups fell to 165 and 115 patients, with 55 and 42 deaths by 28 days, respectively, limiting precision; corresponding interactions were not statistically significant. Three-level analyses did not show a monotonic gradient (Supplementary Table S1, Panels C1,C2).

Most patients had one eligible TTE (1,856/1,976, 93.9%). The first TTE occurred a median of 18.47 h after ICU admission. Among 1,030 patients who received vasopressors before the landmark, 823 underwent their first TTE after vasopressor initiation, and 588 were receiving vasopressors at the time of TTE. Interval midpoints supplied 74.3% of first LVEF values; 47.7% were multiples of 5 and 27.7% were multiples of 10 (Supplementary Table S2, Panel C1). These findings indicated limited numeric precision and potential sensitivity to loading conditions and treatment timing.

### PNI timing, model specification, and precision

When PNI required albumin and lymphocyte count from the same calendar day, 1,552 patients remained and the interaction estimates were attenuated and statistically inconclusive at 28 days (HR, 0.90; 95% CI, 0.79–1.03; *p* = 0.127) and 90 days (HR, 0.92; 95% CI, 0.81–1.04; *p* = 0.165). Requiring an absolute measurement interval of no more than 24 h retained 1,827 patients and produced interaction HRs of 0.83 (95% CI, 0.74–0.94; *p* = 0.002) and 0.86 (95% CI, 0.77–0.96; *p* = 0.006).

The interaction estimates were also similar after omitting mechanical ventilation and vasopressor use (0.82 at 28 days and 0.85 at 90 days; Supplementary Table S1, Panel A2). Adding APS III and pre-landmark inotrope use yielded interaction HRs of 0.81 (95% CI, 0.73–0.90; *p* < 0.001) and 0.84 (95% CI, 0.76–0.93; *p* < 0.001). Exploratory E-values for the point estimate and confidence bound closest to the null were 1.57 and 1.34 at 28 days and 1.50 and 1.27 at 90 days, respectively. An interaction-aware imputation sensitivity produced no logged events and left the fully adjusted interaction HRs unchanged at 0.82 (95% CI, 0.73–0.91; *p* < 0.001) for 28-day mortality and 0.84 (95% CI, 0.76–0.94; *p* = 0.001) for 90-day mortality (Supplementary Table S1, Panel A2).

Model 3 estimated 16 parameters, corresponding to 31.6 and 39.6 events per parameter in the full cohort at 28 and 90 days. Using only the 80 and 96 SCM-stratum events as a conservative denominator gave 5.0 and 6.0 events per parameter. Model 2 estimated 6 parameters and yielded 13.3 and 16.0 SCM-stratum events per parameter, with interaction estimates similar to Model 3. Variance inflation factors were below 2 (maximum, 1.51), and model-term correlations were low (Supplementary Table S2, Panel A2; Supplementary Figure S1).

### Component analyses of PNI

Exploratory component analyses showed different patterns for albumin and lymphocyte count (Supplementary Table S1, Panel D1). Lower albumin was associated with higher mortality without SCM, but albumin-by-SCM interactions were not significant at 28 or 90 days. Lower lymphocyte count showed interactions with SCM at both horizons (interaction HR, 0.89; *p* = 0.001 and *p* < 0.001, respectively), and the lymphocyte interaction remained in joint albumin-plus-lymphocyte models. Albumin and PNI had similar discrimination, whereas lymphocyte count alone added little discrimination without SCM; all marker-related C-index increments were small in the SCM stratum (Supplementary Table S1, Panel D2). These analyses described signal partitioning and did not establish mediation or mechanism.

### External exploratory analysis

In the single-center external cohort, the fully adjusted Firth model yielded an OR of 1.03 (95% CI, 0.79–1.32; *p* = 0.847) per 5-point lower PNI without SCM and 0.91 (95% CI, 0.55–1.49; *p* = 0.704) with LVEF-defined SCM. The ratio of ORs was 0.89 (95% CI, 0.51–1.55; *p* = 0.669; Supplementary Table S2, Panel B1). Although the point estimate was below 1, the wide confidence interval was compatible with materially different interaction effects in either direction. This analysis was statistically inconclusive and was not interpreted as external validation.

## Discussion

### Principal finding

In this selected cohort of 72-h Sepsis-3 survivors with clinically indicated pre-landmark echocardiography and measurable early PNI, the association between lower PNI and mortality differed according to LVEF-defined SCM status. The prespecified primary 28-day analysis showed higher mortality with lower PNI among patients without SCM, whereas no comparable gradient was detected with LVEF-defined SCM. The secondary 90-day analysis, absolute-risk contrasts, and PH-independent RMST analyses supported attenuation in the SCM stratum. However, the finding was sensitive to synchronous PNI measurement and some LVEF definitions. It should therefore be interpreted as phenotype-specific prognostic association rather than as a causal or universally stable effect.

### Interpreting PNI in sepsis

These findings refine how PNI should be interpreted in sepsis. PNI was originally developed as a prognostic index in gastrointestinal surgery (7). In acute critical illness, its interpretation as a purely nutritional measure is limited. Contemporary malnutrition frameworks recognize inflammation and disease burden as etiologic dimensions of malnutrition (17, 18). ESPEN recommends clinical assessment of malnutrition in ICU patients (2). The NUTRIC score combines clinical and illness-severity factors (19), while SCCM/ASPEN guidelines advise against relying on serum protein levels as markers of nutritional status (20). Albumin is affected by inflammation, vascular permeability, hepatic synthesis, dilution, and catabolism (8, 21). Lymphopenia is common in sepsis (9). Reviews of sepsis-induced immunosuppression describe lymphocyte apoptosis and functional impairment (22, 23). Early PNI is therefore better understood as a nutrition-related immune-inflammatory composite marker than as a direct measure of nutritional status. Prior sepsis cohorts primarily estimated overall PNI-mortality associations (10, 11, 24), and another study evaluated PNI alongside other nutrition indices (25). Although one cohort explored other clinical subgroups (24), effect modification by LVEF-defined SCM remained untested. Our results suggest that such uniform interpretation may obscure heterogeneity by early cardiac phenotype.

### Relation to prior work

Three retrospective sepsis cohorts reported associations between lower PNI and higher mortality (10, 11, 24). A systematic review and meta-analysis (12) and an observational study (25) compared PNI with other objective nutritional indices. These studies generally did not test whether the mortality association differed by early cardiac phenotype. Our finding in the non-SCM stratum is directionally consistent with prior sepsis studies, whereas the attenuated association in the SCM stratum adds a cardiac-phenotype qualification to that literature. The present question is also distinct from our previous analysis of PNI as an antecedent marker associated with SCM development (13). Here, cardiac phenotype was defined before follow-up, and the analysis asked whether the subsequent PNI-mortality association differed between strata.

### Cardiac phenotype and early risk

LVEF-defined SCM may shift early risk toward cardiac and hemodynamic factors. An early clinical series documented reversible myocardial depression in septic shock (4). Ventricular function and its assessment are influenced by loading conditions, and treatment is directed toward restoring tissue perfusion (26). A clinical review describes supportive care for cardiogenic shock when present (27), including vasopressors or inotropes as appropriate (28). A comprehensive review describes inflammatory mediators, mitochondrial dysfunction, altered calcium handling, and adrenergic hyporesponsiveness as proposed contributors to septic cardiomyopathy (28). These processes are not captured by nutritional reserve alone. Without overt systolic dysfunction, lower PNI may reflect inflammation-related hypoalbuminemia (8) and sepsis-associated lymphopenia (9), rather than nutritional status alone. With LVEF-defined SCM, the incremental prognostic signal from PNI may be reduced by more immediate cardiac-hemodynamic risk. This explanation is plausible but remains inferential.

### PNI component signals

The exploratory component analyses were compatible with different contributions from albumin and lymphocyte count. Albumin accounted for much of the overall prognostic discrimination, consistent with its role as a negative acute-phase reactant (8, 21). The interaction signal was more closely aligned with lymphocyte count. Sepsis-related lymphopenia can involve apoptosis (9); immune-cell dysfunction has been described in a review (22), and an autopsy study documented lymphocyte depletion in patients who died of sepsis (29). However, component decomposition is not mediation analysis. It does not establish lymphocytes as a causal driver, and no component fully reproduced the combined behavior of PNI.

### Analytic interpretation

The 72-h landmark established PNI and cardiac phenotype before mortality follow-up and reduced immortal-time bias (15, 16). The primary inferential claim rested on one prespecified 28-day interaction test. The 90-day, absolute-risk, RMST, spline, component, selection-weighted, and external analyses were secondary, supportive, or exploratory, and their *p* values were nominal. Relative- and absolute-scale contrasts showed similar attenuation (30), while RMST analyses provided support without requiring proportional hazards. Because the proportional-hazards assumption was not fully satisfied, fixed Cox HRs were treated as model-based summaries over each horizon rather than time-constant effects; RMST served as the principal PH-independent supportive analysis. Pointwise time-varying estimates appeared stronger early, but the formal time trends were not significant. The apparent change in direction after day 28 may reflect depletion of susceptibles and survivor selection, together with fewer late events and reduced precision, rather than a biological reversal.

### Clinical interpretation

The results argue against applying a single PNI interpretation across all septic patients. Lower PNI may identify nutrition-related immune-inflammatory vulnerability among patients without LVEF-defined SCM in our cohort. Earlier sepsis cohorts reported overall PNI-mortality associations (10, 11), while a meta-analysis assessed PNI alongside other objective nutritional indices (12). With LVEF-defined SCM, its incremental association with mortality appeared attenuated, and risk interpretation may need to emphasize cardiac phenotype and hemodynamic status. PNI is not a direct measure of malnutrition, and this study does not support using PNI alone to diagnose malnutrition or trigger nutritional intervention.

### Primary limitations

First, the analytic cohort was restricted to clinically selected patients who underwent TTE. Approximately 73% of landmark survivors lacked eligible pre-landmark LVEF data. Both the direct comparisons and selection models showed material differences between included and excluded patients. Selection weighting balanced measured variables and preserved the interaction estimate, but LVEF-defined SCM remained unobservable without TTE, and weighting could not address unmeasured indications for cardiac imaging. The findings therefore apply most directly to cardiovascularly evaluated 72-h survivors and should not be generalized to all patients with sepsis. The landmark design also excludes deaths before 72 h.

Second, SCM was defined from clinical LVEF measurements. This operational phenotype does not capture diastolic dysfunction, right ventricular dysfunction, strain abnormalities, or the full load-dependent spectrum of septic cardiomyopathy (28). LVEF was commonly measured after vasoactive treatment began and was often derived from interval midpoints or rounded values. Estimates weakened with the first-TTE definition and were not significant at stricter thresholds, without a monotonic gradient; the smaller definition-positive groups and fewer events at those thresholds also reduced precision. Classification may therefore depend on measurement timing, treatment state, numeric precision, and threshold choice. Diagnosis codes recorded during the index hospitalization may also incompletely exclude chronic or ischemic cardiac dysfunction.

### Measurement, modeling, and external-validation limits

Albumin and lymphocyte count were selected independently within the early window. Requiring same-calendar-day measurements reduced the cohort and attenuated the interaction, whereas a 24-h pairing rule was closer to the primary result. This sensitivity may reflect measurement timing, selection, or reduced precision and requires validation with standardized synchronous sampling. PNI was measured once, so trajectories were not evaluated. Fluid balance, glucocorticoid exposure, infection source, a validated septic-shock indicator, and detailed vasoactive doses were unavailable. Mechanical ventilation and vasopressor use may act as both severity markers and potential mediators; models excluding them were similar, but formal mediation was not assessed.

Model 3 had adequate events per parameter in the full cohort, but the conservative SCM-stratum calculation indicated limited precision. The modest E-values also show that residual confounding could move the estimates toward the null. MIMIC-IV mortality records may miss deaths not captured after discharge. Finally, the external cohort differed in case mix, study design (including the absence of a 72-h landmark requirement), sepsis ascertainment, LVEF definition, and outcome. Its in-hospital mortality endpoint depended on length of stay and discharge practices; discharge alive precluded subsequent in-hospital death and would constitute a competing event in a time-to-event analysis. The interaction estimate was statistically inconclusive and cannot be considered formal validation. These limitations preclude causal interpretation and support prospective evaluation with standardized cardiac phenotyping and synchronized repeated immune-inflammatory measurements.

## Conclusion

Among selected 72-h Sepsis-3 survivors with clinically indicated pre-landmark TTE-LVEF assessment and measurable early PNI, lower PNI was associated with higher 28-day mortality without LVEF-defined SCM, whereas this association was attenuated with the prespecified LVEF-defined phenotype. Secondary relative-scale, absolute-risk, and PH-independent RMST analyses supported this pattern, but sensitivity to measurement pairing, LVEF definition, cohort selection, and limited external evidence constrains inference. PNI should be interpreted as a phenotype-dependent nutrition-related immune-inflammatory marker and should not be used alone for nutritional or clinical decision-making.

## Data Availability

The MIMIC-IV database is publicly available through PhysioNet to credentialed users who complete the required training and data use agreement. The single-center external exploratory cohort contains institutional clinical data and is not publicly available due to privacy and ethical restrictions. The analysis code used in this study is publicly available at: https://github.com/shuaiwang619-bot/pni-scm-mortality. Aggregated external cohort data may be provided by the corresponding author upon reasonable request, where permitted by institutional and ethical regulations. No patient-level MIMIC-IV or identifiable external cohort data will be uploaded to GitHub.

## Supplementary material

SUPPLEMENTARY FIGURE S1 Model collinearity diagnostics. Panel A shows variance inflation factors (VIFs) for the 16 terms in the fully adjusted 28-day Cox model fitted in the first completed imputed dataset; the solid and dashed vertical lines mark VIF = 1 and the conventional reference value of 5, respectively. Panel B shows the lower-triangle Spearman correlation matrix for the same model terms in that dataset; diagonal cells are shaded light gray, and coefficients are printed only when absolute rho ≥ 0.25. LVEF, albumin, and lymphocyte count were not included in the mortality model. SCM denotes LVEF-defined septic cardiomyopathy. BMI, body mass index; CAD, coronary artery disease; CKD, chronic kidney disease; COPD, chronic obstructive pulmonary disease; DM, diabetes mellitus; HTN, hypertension; LVEF, left ventricular ejection fraction; MV, mechanical ventilation; PNI, prognostic nutritional index; SOFA, Sequential Organ Failure Assessment; VIF, variance inflation factor.

SUPPLEMENTARY FIGURE S2 Time-varying and restricted mean survival time analyses of the PNI-by-LVEF-defined SCM interaction. Panels A and B show Rubin-pooled interaction HRs (SCM versus No SCM) from fully adjusted time-varying coefficient Cox models at prespecified days after the 72-hour landmark. Lines connect estimates only as visual aids and do not represent an independently fitted trend; shaded areas are pointwise 95% CIs. The formal log-time interaction trends were not significant for the 28-day or 90-day analysis (*p* = 0.364 and *p* = 0.126, respectively). Panels C and D show the change in RMST per 5-point lower PNI at 28 and 90 days within each SCM stratum and the between-stratum difference (SCM minus No SCM). Negative values indicate shorter restricted survival and positive values indicate longer restricted survival. Time-varying Cox models used *g*(*t*) = log(1 + *t*) − log(29) for lower PNI, the PNI-by-SCM interaction, and covariates with PH deviations. RMST estimates were obtained from identity-link linear models with HC0 robust covariance. All models used the covariates listed for Figure 2. Models were fitted across 10 imputed datasets and combined using Rubin's rules. CI, confidence interval; HR, hazard ratio; LVEF, left ventricular ejection fraction; PH, proportional hazards; PNI, prognostic nutritional index; RMST, restricted mean survival time; SCM, septic cardiomyopathy.
