# Supplementary material for: LVEF-defined septic cardiomyopathy modifies the association between the prognostic nutritional index and mortality in adults with sepsis: a 72-h landmark analysis
Source: Front Nutr. 2026 Oct 6;13:1903225. doi: 10.3389/fnut.2026.1903225 (PMC13680185; doi:10.3389/fnut.2026.1903225)
Supplement: Supplementary file 1 [file Table_1.docx]

**Supplementary Table S1. Association, sensitivity, and component analyses of PNI according to LVEF-defined SCM status**

**Panel A. Primary and model-specification sensitivity analyses**

**A1. Primary Cox models**

| **Outcome** | **Model** | **N** | **Events** | **No SCM HR (95% CI)** | **P value** | **LVEF-defined SCM HR (95% CI)** | **P value** | **Ratio of HRs (95% CI)** | **Interaction P value** |
| --- | --- | --- | --- | --- | --- | --- | --- | --- | --- |
| 28-day mortality | Model 1: unadjusted | 1,976 | 506 | 1.20 (1.13–1.27) | <0.001 | 0.97 (0.88–1.07) | 0.580 | 0.81 (0.72–0.91) | <0.001 |
| 28-day mortality | Model 2: age, sex, BMI | 1,976 | 506 | 1.21 (1.14–1.28) | <0.001 | 0.98 (0.89–1.08) | 0.700 | 0.81 (0.72–0.91) | <0.001 |
| 28-day mortality | Model 3: fully adjusted | 1,976 | 506 | 1.18 (1.11–1.24) | <0.001 | 0.96 (0.87–1.05) | 0.384 | 0.82 (0.73–0.91) | <0.001 |
| 90-day mortality | Model 1: unadjusted | 1,976 | 633 | 1.20 (1.14–1.26) | <0.001 | 1.01 (0.92–1.11) | 0.782 | 0.84 (0.76–0.94) | 0.002 |
| 90-day mortality | Model 2: age, sex, BMI | 1,976 | 633 | 1.21 (1.15–1.28) | <0.001 | 1.02 (0.93–1.12) | 0.665 | 0.84 (0.76–0.94) | 0.002 |
| 90-day mortality | Model 3: fully adjusted | 1,976 | 633 | 1.18 (1.12–1.24) | <0.001 | 1.00 (0.91–1.09) | 0.922 | 0.84 (0.76–0.94) | 0.001 |

A1 notes: HRs are reported per 5-point lower PNI. Model 1 was unadjusted; Model 2 adjusted for age, sex, and BMI; Model 3 additionally adjusted for SOFA score, log-transformed lactate and creatinine, coronary artery disease, diabetes mellitus, hypertension, chronic kidney disease, chronic obstructive pulmonary disease, invasive mechanical ventilation, and vasopressor use. Estimates from 10 imputed datasets were combined using Rubin's rules; interaction P values are two-sided Wald t tests with Barnard-Rubin degrees of freedom. No SCM included 1,736 patients with 426 and 537 deaths by 28 and 90 days, respectively; LVEF-defined SCM included 240 patients with 80 and 96 deaths. BMI, body mass index; CI, confidence interval; HR, hazard ratio; LVEF, left ventricular ejection fraction; PNI, prognostic nutritional index; SCM, septic cardiomyopathy; SOFA, Sequential Organ Failure Assessment. Analysis hierarchy: the fully adjusted 28-day interaction is the primary analysis; the corresponding 90-day analysis is secondary and supportive. Panels A2–A5 and C are supportive sensitivity analyses, whereas Panels B and D are exploratory.

**Supplementary Table S1. Association, sensitivity, and component analyses of PNI according to LVEF-defined SCM status (continued)**

**A2. Alternative covariate and imputation specifications**

| **Outcome** | **Model** | **N** | **Events** | **No SCM HR (95% CI)** | **P** | **LVEF-defined SCM HR (95% CI)** | **P** | **Ratio of HRs (95% CI)** | **Interaction P** |
| --- | --- | --- | --- | --- | --- | --- | --- | --- | --- |
| 28-day mortality | Full model without organ support | 1,976 | 506 | 1.17 (1.11–1.24) | <0.001 | 0.96 (0.87–1.05) | 0.351 | 0.82 (0.73–0.91) | <0.001 |
| 28-day mortality | Demographics and comorbidities | 1,976 | 506 | 1.20 (1.13–1.27) | <0.001 | 0.97 (0.88–1.07) | 0.516 | 0.81 (0.72–0.91) | <0.001 |
| 90-day mortality | Full model without organ support | 1,976 | 633 | 1.17 (1.12–1.23) | <0.001 | 0.99 (0.91–1.09) | 0.877 | 0.85 (0.76–0.94) | 0.001 |
| 90-day mortality | Demographics and comorbidities | 1,976 | 633 | 1.20 (1.14–1.26) | <0.001 | 1.01 (0.91–1.11) | 0.916 | 0.84 (0.75–0.93) | 0.001 |
| 28-day mortality | Interaction-aware imputation | 1,976 | 506 | 1.18 (1.11–1.25) | <0.001 | 0.96 (0.87–1.05) | 0.377 | 0.82 (0.73–0.91) | <0.001 |
| 90-day mortality | Interaction-aware imputation | 1,976 | 633 | 1.18 (1.12–1.24) | <0.001 | 1.00 (0.91–1.09) | 0.916 | 0.84 (0.76–0.94) | 0.001 |

A2 notes: HRs are per 5-point lower PNI. The model without organ support omitted invasive mechanical ventilation and vasopressor use from Model 3. The demographics-and-comorbidities model included age, sex, BMI, coronary artery disease, diabetes mellitus, hypertension, chronic kidney disease, and chronic obstructive pulmonary disease. The interaction-aware imputation sensitivity added the centered PNI-by-SCM product as a complete predictor of BMI, lactate, and WBC while retaining complete albumin and lymphocyte components; deterministic PNI was omitted as a redundant predictor. The sensitivity chains used 10 datasets, 20 iterations, and seed 2025, produced no logged events, and left the interaction estimates unchanged at the reported precision. Pooling and interaction testing followed the procedures described for Panel A1.

**Supplementary Table S1. Association, sensitivity, and component analyses of PNI according to LVEF-defined SCM status (continued)**

**A3. Winsorized PNI**

| **Outcome** | **PNI handling** | **N** | **Events** | **No SCM HR (95% CI)** | **LVEF-defined SCM HR (95% CI)** | **Ratio of HRs (95% CI)** | **Linear interaction P** | **Nonlinearity P** | **Spline interaction P** |
| --- | --- | --- | --- | --- | --- | --- | --- | --- | --- |
| 28-day mortality | Original PNI | 1976 | 506 | 1.18 (1.11–1.24) | 0.96 (0.87–1.05) | 0.82 (0.73–0.91) | <0.001 | <0.001 | 0.008 |
| 28-day mortality | Winsorized PNI | 1976 | 506 | 1.20 (1.13–1.27) | 0.98 (0.89–1.09) | 0.82 (0.73–0.92) | <0.001 | 0.140 | 0.044 |
| 90-day mortality | Original PNI | 1976 | 633 | 1.18 (1.12–1.24) | 1.00 (0.91–1.09) | 0.84 (0.76–0.94) | 0.001 | <0.001 | 0.011 |
| 90-day mortality | Winsorized PNI | 1976 | 633 | 1.20 (1.14–1.26) | 1.02 (0.93–1.13) | 0.85 (0.77–0.95) | 0.005 | 0.149 | 0.102 |

A3 notes: PNI was winsorized at the 1st and 99th percentiles (17.45 and 68.50); no patients were excluded, and 19 observations at each tail were capped. HRs are per 5-point lower PNI from fully adjusted Cox models. Linear estimates and interaction P values used the pooling and Wald-testing procedures described for Panel A1; nonlinearity and spline-interaction P values were obtained from Rubin D1 joint tests.

**Supplementary Table S1. Association, sensitivity, and component analyses of PNI according to LVEF-defined SCM status (continued)**

**A4. Additional covariate adjustment and E-value sensitivity analyses**

| **Outcome** | **Model** | **N** | **Events** | **Ratio of HRs (95% CI)** | **Interaction P** | **E-value, point estimate** | **E-value, CI limit nearest null** |
| --- | --- | --- | --- | --- | --- | --- | --- |
| 28-day mortality | Model 3 | 1,976 | 506 | 0.82 (0.73–0.91) | <0.001 | 1.57 | 1.34 |
| 28-day mortality | Model 3 + APS III | 1,976 | 506 | 0.81 (0.73–0.90) | <0.001 | – | – |
| 28-day mortality | Model 3 + inotrope | 1,976 | 506 | 0.82 (0.74–0.92) | <0.001 | – | – |
| 28-day mortality | Model 3 + APS III + inotrope | 1,976 | 506 | 0.81 (0.73–0.90) | <0.001 | – | – |
| 90-day mortality | Model 3 | 1,976 | 633 | 0.84 (0.76–0.94) | 0.001 | 1.50 | 1.27 |
| 90-day mortality | Model 3 + APS III | 1,976 | 633 | 0.83 (0.75–0.92) | <0.001 | – | – |
| 90-day mortality | Model 3 + inotrope | 1,976 | 633 | 0.85 (0.77–0.94) | 0.002 | – | – |
| 90-day mortality | Model 3 + APS III + inotrope | 1,976 | 633 | 0.84 (0.76–0.93) | <0.001 | – | – |

A4 notes: Models were fitted across 10 imputed datasets and combined using Rubin's rules; interaction P values used two-sided Wald t tests with Barnard-Rubin degrees of freedom. APS III and any dobutamine or milrinone use before the 72-hour landmark were added as specified; 40 patients received an inotrope before the landmark. E-values were calculated only for the primary Model 3 interaction estimates and are exploratory HR-scale sensitivity measures; they do not remove residual confounding or support causal interpretation. APS III, Acute Physiology Score III; CI, confidence interval; HR, hazard ratio.

**Supplementary Table S1. Association, sensitivity, and component analyses of PNI according to LVEF-defined SCM status (continued)**

**A5. PNI measurement-pairing sensitivity analyses**

| **Outcome** | **Pairing rule** | **N** | **SCM n** | **Events** | **No SCM HR (95% CI)** | **LVEF-defined SCM HR (95% CI)** | **Ratio of HRs (95% CI)** | **Interaction P** |
| --- | --- | --- | --- | --- | --- | --- | --- | --- |
| 28-day mortality | Original pairing rule | 1,976 | 240 | 506 | 1.18 (1.11–1.24) | 0.96 (0.87–1.05) | 0.82 (0.73–0.91) | <0.001 |
| 28-day mortality | Same calendar date | 1,552 | 182 | 409 | 1.12 (1.05–1.20) | 1.01 (0.90–1.14) | 0.90 (0.79–1.03) | 0.127 |
| 28-day mortality | Absolute albumin-lymphocyte gap ≤24 h | 1,827 | 222 | 464 | 1.15 (1.09–1.22) | 0.96 (0.87–1.06) | 0.83 (0.74–0.94) | 0.002 |
| 90-day mortality | Original pairing rule | 1,976 | 240 | 633 | 1.18 (1.12–1.24) | 1.00 (0.91–1.09) | 0.84 (0.76–0.94) | 0.001 |
| 90-day mortality | Same calendar date | 1,552 | 182 | 519 | 1.13 (1.07–1.20) | 1.04 (0.93–1.16) | 0.92 (0.81–1.04) | 0.165 |
| 90-day mortality | Absolute albumin-lymphocyte gap ≤24 h | 1,827 | 222 | 583 | 1.16 (1.10–1.23) | 1.00 (0.91–1.10) | 0.86 (0.77–0.96) | 0.006 |

A5 notes: PNI was recalculated using the original pairing rule, the best same-calendar-date albumin-lymphocyte pair, or the best pair with an absolute sampling-time gap of 24 hours or less. Fully adjusted Model 3 Cox models were fitted in 10 imputed datasets with 20 MICE iterations and pooled using Rubin's rules. HRs are per 5-point lower PNI. CI, confidence interval; HR, hazard ratio; LVEF, left ventricular ejection fraction; PNI, prognostic nutritional index; SCM, septic cardiomyopathy.

**Supplementary Table S1. Association, sensitivity, and component analyses of PNI according to LVEF-defined SCM status (continued)**

**Panel B. Nonlinearity and absolute-risk translation**

**B1. Spline-model tests**

| **Outcome** | **Diagnostic term** | **Method** | **P value or range** |
| --- | --- | --- | --- |
| 28-day mortality | PNI nonlinearity beyond the linear interaction model (Original PNI) | Rubin D1 joint test | <0.001 |
| 28-day mortality | PNI spline x SCM interaction (Original PNI) | Rubin D1 joint test | 0.008 |
| 28-day mortality | PNI nonlinearity beyond the linear interaction model (Winsorized PNI (1st/99th percentile)) | Rubin D1 joint test | 0.140 |
| 28-day mortality | PNI spline x SCM interaction (Winsorized PNI (1st/99th percentile)) | Rubin D1 joint test | 0.044 |
| 90-day mortality | PNI nonlinearity beyond the linear interaction model (Original PNI) | Rubin D1 joint test | <0.001 |
| 90-day mortality | PNI spline x SCM interaction (Original PNI) | Rubin D1 joint test | 0.011 |
| 90-day mortality | PNI nonlinearity beyond the linear interaction model (Winsorized PNI (1st/99th percentile)) | Rubin D1 joint test | 0.149 |
| 90-day mortality | PNI spline x SCM interaction (Winsorized PNI (1st/99th percentile)) | Rubin D1 joint test | 0.102 |

B1 notes: Nonlinearity and spline-interaction comparisons were pooled across 10 imputed datasets using Rubin D1 joint tests.

**Supplementary Table S1. Association, sensitivity, and component analyses of PNI according to LVEF-defined SCM status (continued)**

**B2. Standardized absolute-risk translation**

| **Outcome** | **SCM** | **Standardization population N** | **Events** | **Adjusted risk at PNI 40, % (95% CI)** | **Adjusted risk at PNI 30, % (95% CI)** | **Risk difference, percentage points (95% CI)** |
| --- | --- | --- | --- | --- | --- | --- |
| 28-day mortality | No SCM | 1976 | 506 | 21.7 (19.2 to 24.0) | 28.3 (25.8 to 31.0) | 6.6 (4.1 to 9.3) |
| 28-day mortality | SCM | 1976 | 506 | 32.3 (26.3 to 38.5) | 31.3 (25.0 to 38.1) | -1.0 (-5.2 to 4.6) |
| 90-day mortality | No SCM | 1976 | 633 | 27.6 (25.0 to 30.1) | 35.5 (32.7 to 38.3) | 7.9 (4.9 to 11.1) |
| 90-day mortality | SCM | 1976 | 633 | 38.6 (31.9 to 44.9) | 39.6 (32.1 to 46.4) | 1.0 (-3.4 to 7.0) |

**B3. Absolute-scale interaction contrast**

| **Outcome** | **Contrast** | **Risk-difference contrast, percentage points (95% CI)** |
| --- | --- | --- |
| 28-day mortality | Risk difference in SCM minus risk difference in No SCM | -7.6 (-12.9 to -1.8) |
| 90-day mortality | Risk difference in SCM minus risk difference in No SCM | -6.9 (-12.5 to -0.3) |

B2-B3 notes: These estimates came from separate fully adjusted fixed-time logistic models, not from the Cox spline curves. Risks were standardized over the analytic-cohort covariate distribution after setting PNI to 30 or 40 and SCM status to each stratum. Risk differences were calculated as risk at PNI 30 minus risk at PNI 40; percentile 95% CIs were obtained from 1,000 nonparametric bootstrap resamples (seed 2025). CI, confidence interval; PNI, prognostic nutritional index; SCM, septic cardiomyopathy. The reported N=1,976 is the covariate standardization population for each counterfactual SCM state, not the number observed in that stratum.

**Supplementary Table S1. Association, sensitivity, and component analyses of PNI according to LVEF-defined SCM status (continued)**

**Panel C. LVEF-definition and non-proportional-hazards sensitivity analyses**

**C1. Binary LVEF definitions**

| **Outcome** | **LVEF definition** | **Definition negative, n/events** | **PNI HR (95% CI)** | **Definition positive, n/events** | **PNI HR (95% CI)** | **Ratio of HRs (95% CI)** | **P value** |
| --- | --- | --- | --- | --- | --- | --- | --- |
| 28-day | Primary LVEF-defined SCM | 1,736/426 | 1.18 (1.11–1.24) | 240/80 | 0.96 (0.87–1.05) | 0.82 (0.73–0.91) | <0.001 |
| 28-day | Any eligible LVEF <50% | 1,739/426 | 1.18 (1.11–1.25) | 237/80 | 0.96 (0.87–1.05) | 0.82 (0.73–0.91) | <0.001 |
| 28-day | Any eligible LVEF <45% | 1,811/451 | 1.13 (1.07–1.19) | 165/55 | 1.08 (0.94–1.24) | 0.96 (0.83–1.10) | 0.534 |
| 28-day | Any eligible LVEF <40% | 1,861/464 | 1.14 (1.08–1.20) | 115/42 | 1.02 (0.88–1.18) | 0.89 (0.77–1.04) | 0.156 |
| 28-day | First eligible TTE LVEF <50% | 1,754/429 | 1.15 (1.09–1.22) | 222/77 | 1.01 (0.92–1.12) | 0.88 (0.78–0.99) | 0.027 |
| 28-day | First eligible TTE LVEF <45% | 1,818/453 | 1.13 (1.07–1.19) | 158/53 | 1.09 (0.95–1.25) | 0.96 (0.83–1.12) | 0.625 |
| 28-day | First eligible TTE LVEF <40% | 1,866/467 | 1.14 (1.08–1.20) | 110/39 | 1.02 (0.87–1.18) | 0.89 (0.76–1.05) | 0.171 |
| 90-day | Primary LVEF-defined SCM | 1,736/537 | 1.18 (1.12–1.24) | 240/96 | 1.00 (0.91–1.09) | 0.84 (0.76–0.94) | 0.001 |
| 90-day | Any eligible LVEF <50% | 1,739/537 | 1.18 (1.12–1.24) | 237/96 | 0.99 (0.91–1.09) | 0.84 (0.76–0.93) | 0.001 |
| 90-day | Any eligible LVEF <45% | 1,811/564 | 1.14 (1.09–1.20) | 165/69 | 1.12 (0.99–1.26) | 0.98 (0.86–1.11) | 0.704 |
| 90-day | Any eligible LVEF <40% | 1,861/583 | 1.15 (1.10–1.21) | 115/50 | 1.05 (0.92–1.21) | 0.91 (0.79–1.06) | 0.223 |
| 90-day | First eligible TTE LVEF <50% | 1,754/541 | 1.16 (1.10–1.22) | 222/92 | 1.05 (0.95–1.16) | 0.91 (0.81–1.01) | 0.075 |
| 90-day | First eligible TTE LVEF <45% | 1,818/567 | 1.14 (1.09–1.20) | 158/66 | 1.12 (0.99–1.27) | 0.98 (0.86–1.12) | 0.797 |
| 90-day | First eligible TTE LVEF <40% | 1,866/586 | 1.15 (1.10–1.21) | 110/47 | 1.06 (0.92–1.22) | 0.92 (0.79–1.06) | 0.259 |

**Supplementary Table S1. Association, sensitivity, and component analyses of PNI according to LVEF-defined SCM status (continued)**

**C2. Three-level LVEF analyses**

| **Outcome** | **LVEF summary** | **≥50%, n/events; PNI HR (95% CI)** | **40%-49%, n/events; PNI HR (95% CI)** | **<40%, n/events; PNI HR (95% CI)** | **D1 joint interaction test** | **P value** |
| --- | --- | --- | --- | --- | --- | --- |
| 28-day | First eligible TTE | 1,754/429; 1.15 (1.09–1.22) | 112/38; 1.01 (0.88–1.16) | 110/39; 1.02 (0.87–1.18) | F(2,1955.1)=2.47 | 0.085 |
| 28-day | Lowest eligible LVEF | 1,739/426; 1.18 (1.11–1.25) | 122/38; 0.92 (0.82–1.03) | 115/42; 1.02 (0.88–1.18) | F(2,1955.7)=7.77 | <0.001 |
| 90-day | First eligible TTE | 1,754/541; 1.16 (1.10–1.22) | 112/45; 1.05 (0.91–1.20) | 110/47; 1.06 (0.92–1.22) | F(2,1954.5)=1.60 | 0.201 |
| 90-day | Lowest eligible LVEF | 1,739/537; 1.18 (1.12–1.24) | 122/46; 0.95 (0.84–1.07) | 115/50; 1.05 (0.92–1.21) | F(2,1955.6)=6.10 | 0.002 |

C1-C2 notes: HRs describe the association per 5-point lower PNI within each LVEF group and were obtained from fully adjusted Cox models across 10 imputed datasets, pooled using Rubin's rules. Binary interaction P values are Rubin-pooled Wald tests; three-level interaction P values are Rubin D1 joint tests. Primary LVEF-defined SCM was any eligible LVEF <50% or a decline >10 percentage points to <55%. CI, confidence interval; HR, hazard ratio; LVEF, left ventricular ejection fraction; PNI, prognostic nutritional index; SCM, septic cardiomyopathy; TTE, transthoracic echocardiography.

**Supplementary Table S1. Association, sensitivity, and component analyses of PNI according to LVEF-defined SCM status (continued)**

**C3. Time-varying coefficient Cox models**

| **Outcome** | **Day** | **Ratio of HRs (95% CI)** | **P value** | **Log-time interaction P value** |
| --- | --- | --- | --- | --- |
| 28-day mortality | 1 | 0.81 (0.70–0.93) | 0.002 | 0.364 |
| 28-day mortality | 7 | 0.86 (0.77–0.97) | 0.013 | 0.364 |
| 28-day mortality | 14 | 0.89 (0.76–1.04) | 0.149 | 0.364 |
| 28-day mortality | 28 | 0.92 (0.74–1.14) | 0.442 | 0.364 |
| 90-day mortality | 1 | 0.80 (0.70–0.91) | 0.001 | 0.126 |
| 90-day mortality | 7 | 0.87 (0.79–0.96) | 0.006 | 0.126 |
| 90-day mortality | 14 | 0.90 (0.80–1.02) | 0.095 | 0.126 |
| 90-day mortality | 28 | 0.94 (0.81–1.10) | 0.451 | 0.126 |
| 90-day mortality | 60 | 0.99 (0.81–1.21) | 0.903 | 0.126 |
| 90-day mortality | 90 | 1.01 (0.80–1.28) | 0.917 | 0.126 |

**C4. Window-specific Cox models and PH diagnostics**

| **Window** | **N/events** | **Ratio of HRs (95% CI)** | **P value** | **Interaction cox.zph P range** | **Global cox.zph P range** |
| --- | --- | --- | --- | --- | --- |
| Days 0–28 | 1,976/506 | 0.82 (0.73–0.91) | <0.001 | 0.011–0.013 | 0.001–0.003 |
| Days 28–90 among 28-day survivors | 1,470/127 | 1.08 (0.81–1.45) | 0.597 | 0.016–0.017 | 0.006–0.032 |

**C5. Adjusted RMST interaction analyses**

| **Outcome/tau** | **Contrast** | **RMST difference, days (95% CI)** | **P value** |
| --- | --- | --- | --- |
| 28 days | PNI per 5-point lower: No SCM | -0.51 (-0.73 to -0.28) | <0.001 |
| 28 days | PNI per 5-point lower: LVEF-defined SCM | 0.34 (-0.22 to 0.90) | 0.229 |
| 28 days | PNI-by-SCM interaction | 0.85 (0.25 to 1.45) | 0.005 |
| 90 days | PNI per 5-point lower: No SCM | -2.42 (-3.32 to -1.52) | <0.001 |
| 90 days | PNI per 5-point lower: LVEF-defined SCM | 0.09 (-1.94 to 2.12) | 0.930 |
| 90 days | PNI-by-SCM interaction | 2.51 (0.31 to 4.71) | 0.025 |

C3-C5 notes: All analyses used the fully adjusted Model 3 covariates across 10 imputed datasets, with estimates combined using Rubin's rules. Time-varying Cox models used g(t) = log(1 + t) - log(29). Window-specific Schoenfeld-residual P values are ranges across imputations and were not Rubin-pooled. Follow-up was complete to each horizon without earlier censoring, so restricted survival time was directly observed. RMST models used HC0 heteroskedasticity-consistent robust covariance; positive interaction contrasts indicate attenuation of the adverse lower-PNI association in the LVEF-defined SCM group. CI, confidence interval; HR, hazard ratio; PH, proportional hazards; PNI, prognostic nutritional index; RMST, restricted mean survival time; SCM, septic cardiomyopathy. Among 28-day survivors in the LVEF-defined SCM stratum, the 28–90-day HR per 5-point lower PNI was 1.30 (95% CI, 0.99–1.70). This survivor-selected estimate is exploratory.

**Supplementary Table S1. Association, sensitivity, and component analyses of PNI according to LVEF-defined SCM status (continued)**

**Panel D. Component-specific analyses and discrimination**

**D1. Component-specific Cox models**

| **Outcome** | **Model** | **Marker** | **N** | **Events** | **No SCM HR (95% CI)** | **P** | **LVEF-defined SCM HR (95% CI)** | **P** | **Ratio of HRs (95% CI)** | **Interaction P** |
| --- | --- | --- | --- | --- | --- | --- | --- | --- | --- | --- |
| 28-day mortality | PNI | PNI, per 5-point lower | 1,976 | 506 | 1.18 (1.11–1.24) | <0.001 | 0.96 (0.87–1.05) | 0.384 | 0.82 (0.73–0.91) | <0.001 |
| 28-day mortality | Albumin | Albumin, per 5 g/L lower | 1,976 | 506 | 1.24 (1.16–1.33) | <0.001 | 1.10 (0.94–1.27) | 0.233 | 0.88 (0.75–1.04) | 0.139 |
| 28-day mortality | Lymphocyte | Lymphocyte, per 0.5 ×10⁹/L lower | 1,976 | 506 | 1.05 (1.00–1.11) | 0.041 | 0.94 (0.89–0.98) | 0.011 | 0.89 (0.83–0.95) | 0.001 |
| 28-day mortality | Albumin + lymphocyte | Albumin, per 5 g/L lower | 1,976 | 506 | 1.23 (1.15–1.33) | <0.001 | 1.12 (0.96–1.30) | 0.141 | 0.91 (0.77–1.07) | 0.249 |
| 28-day mortality | Albumin + lymphocyte | Lymphocyte, per 0.5 ×10⁹/L lower | 1,976 | 506 | 1.04 (0.99–1.09) | 0.140 | 0.93 (0.89–0.98) | 0.007 | 0.90 (0.84–0.97) | 0.003 |
| 90-day mortality | PNI | PNI, per 5-point lower | 1,976 | 633 | 1.18 (1.12–1.24) | <0.001 | 1.00 (0.91–1.09) | 0.922 | 0.84 (0.76–0.94) | 0.001 |
| 90-day mortality | Albumin | Albumin, per 5 g/L lower | 1,976 | 633 | 1.23 (1.16–1.31) | <0.001 | 1.14 (1.00–1.31) | 0.057 | 0.93 (0.80–1.08) | 0.323 |
| 90-day mortality | Lymphocyte | Lymphocyte, per 0.5 ×10⁹/L lower | 1,976 | 633 | 1.06 (1.02–1.11) | 0.007 | 0.94 (0.90–0.99) | 0.029 | 0.89 (0.83–0.95) | <0.001 |
| 90-day mortality | Albumin + lymphocyte | Albumin, per 5 g/L lower | 1,976 | 633 | 1.22 (1.15–1.31) | <0.001 | 1.17 (1.02–1.34) | 0.028 | 0.95 (0.82–1.11) | 0.533 |
| 90-day mortality | Albumin + lymphocyte | Lymphocyte, per 0.5 ×10⁹/L lower | 1,976 | 633 | 1.05 (1.00–1.09) | 0.037 | 0.94 (0.89–0.99) | 0.013 | 0.90 (0.84–0.96) | 0.001 |

D1 notes: Estimates are scaled per 5-point lower PNI, 5 g/L lower albumin, or 0.5 x 10⁹/L lower lymphocyte count. Models used the fully adjusted covariate set. PNI, albumin, and lymphocyte count were fitted separately except in the combined albumin-plus-lymphocyte model. Estimates from 10 imputed datasets were combined using Rubin's rules; interaction P values are two-sided Wald t tests with Barnard-Rubin degrees of freedom. CI, confidence interval; HR, hazard ratio; PNI, prognostic nutritional index; SCM, septic cardiomyopathy.

**Supplementary Table S1. Association, sensitivity, and component analyses of PNI according to LVEF-defined SCM status (continued)**

**D2. SCM-stratified C-index**

| **Outcome** | **SCM stratum** | **N/events** | **Covariates only** | **+ PNI** | **+ Albumin** | **+ Lymphocyte** |
| --- | --- | --- | --- | --- | --- | --- |
| 28-day mortality | No SCM | 1,736/426 | 0.664 (0.639–0.687) | 0.679 (0.654–0.703); Δ +0.015 | 0.679 (0.655–0.703); Δ +0.016 | 0.665 (0.640–0.689); Δ +0.001 |
| 28-day mortality | LVEF-defined SCM | 240/80 | 0.679 (0.620–0.734) | 0.681 (0.622–0.735); Δ +0.002 | 0.679 (0.620–0.733); Δ +0.000 | 0.685 (0.627–0.739); Δ +0.006 |
| 90-day mortality | No SCM | 1,736/537 | 0.663 (0.641–0.685) | 0.681 (0.659–0.703); Δ +0.018 | 0.680 (0.658–0.702); Δ +0.017 | 0.666 (0.644–0.688); Δ +0.003 |
| 90-day mortality | LVEF-defined SCM | 240/96 | 0.662 (0.608–0.711) | 0.663 (0.610–0.712); Δ +0.001 | 0.665 (0.613–0.714); Δ +0.004 | 0.668 (0.614–0.718); Δ +0.006 |

D2 notes: C-index estimates were obtained within each SCM stratum and pooled on the logit scale across 10 imputed datasets. Delta denotes the difference from the covariates-only model within the same outcome and stratum. The SCM stratum contained 240 patients, including 80 and 96 deaths by 28 and 90 days, respectively; estimates in this stratum are therefore less precise. C-index, concordance index; CI, confidence interval; PNI, prognostic nutritional index; SCM, septic cardiomyopathy.
