# Supplementary material for: LVEF-defined septic cardiomyopathy modifies the association between the prognostic nutritional index and mortality in adults with sepsis: a 72-h landmark analysis
Source: Front Nutr. 2026 Oct 6;13:1903225. doi: 10.3389/fnut.2026.1903225 (PMC13680185; doi:10.3389/fnut.2026.1903225)
Supplement: Supplementary file 2 [file Table_2.docx]

**Supplementary Table S2. Model diagnostics, cohort-selection audits, missingness, and external exploratory analysis**

**Panel A. Model diagnostics**

**A1. Proportional-hazards diagnostics**

| **Outcome** | **Diagnostic term** | **Method** | **P value or range** |
| --- | --- | --- | --- |
| 28-day mortality | PNI per 5-point lower | Schoenfeld residual test | 0.003–0.003 |
| 28-day mortality | PNI x SCM interaction | Schoenfeld residual test | 0.011–0.013 |
| 28-day mortality | LVEF-defined SCM | Schoenfeld residual test | 0.292–0.305 |
| 28-day mortality | Age | Schoenfeld residual test | 0.518–0.540 |
| 28-day mortality | Female sex | Schoenfeld residual test | 0.371–0.380 |
| 28-day mortality | Body mass index | Schoenfeld residual test | 0.070–0.393 |
| 28-day mortality | SOFA score | Schoenfeld residual test | 0.596–0.618 |
| 28-day mortality | Log-transformed lactate | Schoenfeld residual test | 0.003–0.022 |
| 28-day mortality | Log-transformed creatinine | Schoenfeld residual test | 0.029–0.032 |
| 28-day mortality | Coronary artery disease | Schoenfeld residual test | 0.593–0.612 |
| 28-day mortality | Diabetes mellitus | Schoenfeld residual test | 0.842–0.859 |
| 28-day mortality | Hypertension | Schoenfeld residual test | 0.106–0.110 |
| 28-day mortality | Chronic kidney disease | Schoenfeld residual test | 0.149–0.156 |
| 28-day mortality | Chronic obstructive pulmonary disease | Schoenfeld residual test | 0.775–0.804 |
| 28-day mortality | Invasive mechanical ventilation | Schoenfeld residual test | 0.114–0.122 |
| 28-day mortality | Vasopressor use | Schoenfeld residual test | 0.157–0.172 |
| 28-day mortality | Global test | Schoenfeld residual test | 0.001–0.003 |
| 90-day mortality | PNI per 5-point lower | Schoenfeld residual test | 0.002–0.003 |
| 90-day mortality | PNI x SCM interaction | Schoenfeld residual test | 0.002–0.002 |
| 90-day mortality | LVEF-defined SCM | Schoenfeld residual test | 0.140–0.148 |
| 90-day mortality | Age | Schoenfeld residual test | 0.606–0.636 |
| 90-day mortality | Female sex | Schoenfeld residual test | 0.166–0.176 |
| 90-day mortality | Body mass index | Schoenfeld residual test | 0.110–0.692 |
| 90-day mortality | SOFA score | Schoenfeld residual test | 0.320–0.333 |
| 90-day mortality | Log-transformed lactate | Schoenfeld residual test | 0.003–0.078 |
| 90-day mortality | Log-transformed creatinine | Schoenfeld residual test | 0.048–0.052 |
| 90-day mortality | Coronary artery disease | Schoenfeld residual test | 0.441–0.458 |
| 90-day mortality | Diabetes mellitus | Schoenfeld residual test | 0.519–0.541 |
| 90-day mortality | Hypertension | Schoenfeld residual test | 0.146–0.153 |
| 90-day mortality | Chronic kidney disease | Schoenfeld residual test | 0.021–0.024 |
| 90-day mortality | Chronic obstructive pulmonary disease | Schoenfeld residual test | 0.554–0.584 |
| 90-day mortality | Invasive mechanical ventilation | Schoenfeld residual test | 0.008–0.009 |
| 90-day mortality | Vasopressor use | Schoenfeld residual test | 0.202–0.225 |
| 90-day mortality | Global test | Schoenfeld residual test | <0.001 |

A1 notes: Proportional-hazards diagnostics were calculated separately in each completed imputed dataset; ranges are shown because Schoenfeld-residual tests do not have a standard Rubin-pooling rule. BMI, body mass index; CAD, coronary artery disease; CKD, chronic kidney disease; COPD, chronic obstructive pulmonary disease; DM, diabetes mellitus; HTN, hypertension; MV, mechanical ventilation; PH, proportional hazards; PNI, prognostic nutritional index; SCM, septic cardiomyopathy; SOFA, Sequential Organ Failure Assessment. Analysis hierarchy: Panels A and D are descriptive diagnostics, Panel B is exploratory, and Panel C is a supportive selection-bias sensitivity analysis.

**Supplementary Table S2. Model diagnostics, cohort-selection audits, missingness, and external exploratory analysis (continued)**

**A2. Variance inflation factors**

| **Variable** | **28-day mortality VIF** | **90-day mortality VIF** |
| --- | --- | --- |
| PNI, per 5-point decrease, centered | 1.25 | 1.25 |
| SCM | 1.08 | 1.08 |
| PNI, per 5-point decrease, centered x SCM | 1.21 | 1.21 |
| Age | 1.32 | 1.32 |
| Female sex | 1.06 | 1.06 |
| BMI | 1.13 | 1.13 |
| SOFA score | 1.32 | 1.32 |
| Log-transformed lactate | 1.21 | 1.21 |
| Log-transformed creatinine | 1.51 | 1.51 |
| Coronary artery disease | 1.17 | 1.17 |
| Diabetes mellitus | 1.13 | 1.13 |
| Hypertension | 1.29 | 1.29 |
| Chronic kidney disease | 1.26 | 1.26 |
| COPD | 1.03 | 1.03 |
| Mechanical ventilation | 1.15 | 1.15 |
| Vasopressor use | 1.21 | 1.21 |

A2 notes: Variance inflation factors were calculated from the fully adjusted model matrix in the first completed imputed dataset after centering the PNI term. This panel is descriptive; all values were below 2. Abbreviations are defined in Panel A1; VIF, variance inflation factor.

**Supplementary Table S2. Model diagnostics, cohort-selection audits, missingness, and external exploratory analysis (continued)**

**Panel B. External exploratory analysis**

**B1. In-hospital death in the independent single-center cohort**

| **Model** | **N** | **Deaths** | **No SCM OR (95% CI)** | **P value** | **LVEF-defined SCM OR (95% CI)** | **P value** | **Ratio of ORs (95% CI)** | **Interaction P value** |
| --- | --- | --- | --- | --- | --- | --- | --- | --- |
| Model 1: unadjusted | 272 | 77 | 1.12 (0.89–1.42) | 0.326 | 0.96 (0.59–1.55) | 0.861 | 0.85 (0.50–1.46) | 0.557 |
| Model 2: age, sex, BMI | 272 | 77 | 1.05 (0.82–1.34) | 0.715 | 0.95 (0.59–1.53) | 0.826 | 0.91 (0.53–1.55) | 0.716 |
| Model 3: limited clinical adjustment | 272 | 77 | 1.03 (0.79–1.32) | 0.847 | 0.91 (0.55–1.49) | 0.704 | 0.89 (0.51–1.55) | 0.669 |

B1 notes: This was an exploratory consistency analysis, not a formal external validation. The cohort included 272 patients and 77 in-hospital deaths: 199 patients without SCM (64 deaths) and 73 patients with LVEF-defined SCM (13 deaths). ORs are per 5-point lower PNI. Model 1 was unadjusted; Model 2 adjusted for age, sex, and BMI; Model 3 additionally adjusted for SOFA score and log-transformed lactate and creatinine. Firth penalized logistic models were fitted in each of 10 imputed datasets; estimates were combined using Rubin's rules, and interaction P values are two-sided Wald t tests with Barnard-Rubin degrees of freedom. BMI, body mass index; CI, confidence interval; LVEF, left ventricular ejection fraction; OR, odds ratio; PNI, prognostic nutritional index; SCM, septic cardiomyopathy; SOFA, Sequential Organ Failure Assessment.

**Supplementary Table S2. Model diagnostics, cohort-selection audits, missingness, and external exploratory analysis (continued)**

**Panel C. Echocardiographic ascertainment and cohort-selection audits**

**C1. LVEF ascertainment, timing, and measurement audit**

| **Metric** | **Result** |
| --- | --- |
| SCM classified by decline criterion alone | 3/240; 0 deaths by 28 or 90 days |
| Eligible TTE examinations | One examination: 1,856/1,976 (93.9%); two or more: 120/1,976 (6.1%); range, 1–6 |
| First TTE relative to ICU admission | Median 18.47 h (IQR, 9.96–37.08); before ICU 109 (5.5%); 0-<24 h 1,089 (55.1%); 24–72 h 778 (39.4%) |
| Vasopressor timing | Used before landmark: 1,030; first TTE at/after treatment start: 823/1,030 (79.9%); vasopressor active at first TTE: 588/1,976 (29.8%) |
| Inotrope timing | Dobutamine or milrinone before landmark: 40; first TTE at/after treatment start: 14/40 (35.0%); inotrope active at first TTE: 8/1,976 (0.4%) |
| First LVEF source | Interval midpoint 1,468 (74.3%); biplane 288 (14.6%); lower bound 154 (7.8%); 3D 34 (1.7%); upper bound 32 (1.6%) |
| LVEF numeric heaping | Exact multiples of 5: 943/1,976 (47.7%); exact multiples of 10: 547/1,976 (27.7%); 76 unique values |

**Supplementary Table S2. Model diagnostics, cohort-selection audits, missingness, and external exploratory analysis (continued)**

**C2. Selection for eligible pre-landmark TTE-LVEF**

| **Characteristic** | **Level** | **No eligible TTE (n=12,407), unweighted** | **Eligible TTE (n=4,669), unweighted** | **Absolute SMD before / after IPSW** |
| --- | --- | --- | --- | --- |
| Age, years | Median [IQR] | 63.00 [52.00, 74.00] | 64.00 [52.00, 75.00] | 0.036 / 0.013 |
| BMI, kg/m² | Median [IQR] | 27.55 [23.99, 31.99] | 27.72 [23.88, 32.74] | 0.061 / 0.001 |
| SOFA score | Median [IQR] | 3.00 [2.00, 4.00] | 3.00 [2.00, 4.00] | 0.115 / 0.004 |
| APS III | Median [IQR] | 41.00 [30.00, 56.00] | 50.00 [37.00, 66.00] | 0.383 / 0.009 |
| Lactate, mmol/L | Median [IQR] | 2.30 [1.60, 3.50] | 2.30 [1.50, 4.10] | 0.147 / 0.006 |
| Creatinine, mg/dL | Median [IQR] | 1.00 [0.80, 1.40] | 1.20 [0.80, 2.00] | 0.206 / 0.014 |
| Female sex | Yes | 4924 (39.7%) | 2079 (44.5%) | 0.098 / 0.016 |
| In hospital at 72 h | Yes | 11902 (95.9%) | 4525 (96.9%) | 0.053 / 0.006 |
| Mechanical ventilation | Yes | 7203 (58.1%) | 2588 (55.4%) | 0.053 / 0.037 |
| Vasopressor use | Yes | 5030 (40.5%) | 2008 (43.0%) | 0.050 / 0.025 |
| Coronary artery disease | Yes | 3400 (27.4%) | 1398 (29.9%) | 0.056 / 0.048 |
| Diabetes mellitus | Yes | 3204 (25.8%) | 1269 (27.2%) | 0.031 / 0.008 |
| Hypertension | Yes | 7400 (59.6%) | 2810 (60.2%) | 0.011 / 0.012 |
| Chronic kidney disease | Yes | 1687 (13.6%) | 787 (16.9%) | 0.091 / 0.013 |
| COPD | Yes | 2414 (19.5%) | 1104 (23.6%) | 0.102 / 0.000 |
| BMI missing | Yes | 1801 (14.5%) | 695 (14.9%) | 0.010 / 0.006 |
| Lactate missing | Yes | 2439 (19.7%) | 841 (18.0%) | 0.042 / 0.031 |
| Creatinine missing | Yes | 31 (0.2%) | 9 (0.2%) | 0.012 / 0.005 |
| Admission type | Elective/surgical | 2563 (20.7%) | 266 (5.7%) | 0.454 / 0.009 |
| Admission type | Emergency/urgent | 8556 (69.0%) | 3818 (81.8%) | 0.301 / 0.003 |
| Admission type | Observation | 1288 (10.4%) | 585 (12.5%) | 0.067 / 0.006 |
| Admission location | Clinician referral | 3903 (31.5%) | 750 (16.1%) | 0.368 / 0.006 |
| Admission location | Emergency room | 4597 (37.1%) | 1987 (42.6%) | 0.113 / 0.002 |
| Admission location | Hospital transfer | 3119 (25.1%) | 1621 (34.7%) | 0.210 / 0.005 |
| Admission location | Other | 428 (3.4%) | 131 (2.8%) | 0.037 / 0.003 |
| Admission location | Self referral | 360 (2.9%) | 180 (3.9%) | 0.053 / 0.001 |
| Race | Asian | 393 (3.2%) | 127 (2.7%) | 0.026 / 0.010 |
| Race | Black | 966 (7.8%) | 402 (8.6%) | 0.030 / 0.006 |
| Race | Hispanic/Latino | 471 (3.8%) | 156 (3.3%) | 0.025 / 0.007 |
| Race | Other | 547 (4.4%) | 212 (4.5%) | 0.006 / 0.008 |
| Race | Unknown | 1829 (14.7%) | 889 (19.0%) | 0.115 / 0.006 |
| Race | White | 8201 (66.1%) | 2883 (61.7%) | 0.091 / 0.009 |
| Insurance | Medicaid | 1888 (15.2%) | 791 (16.9%) | 0.047 / 0.002 |
| Insurance | Medicare | 6031 (48.6%) | 2442 (52.3%) | 0.074 / 0.002 |
| Insurance | Other | 380 (3.1%) | 129 (2.8%) | 0.018 / 0.009 |
| Insurance | Private | 3874 (31.2%) | 1221 (26.2%) | 0.112 / 0.004 |
| Insurance | Unknown | 234 (1.9%) | 86 (1.8%) | 0.003 / 0.001 |

**Supplementary Table S2. Model diagnostics, cohort-selection audits, missingness, and external exploratory analysis (continued)**

**C3. Crude mortality outcomes by TTE-LVEF eligibility**

| **Outcome** | **No eligible TTE, n (%)** | **Eligible TTE, n (%)** | **Unweighted risk difference, percentage points** | **Interpretation** |
| --- | --- | --- | --- | --- |
| Hospital mortality | 1097 (8.8%) | 881 (18.9%) | 10.0 | Descriptive; not a causal effect of TTE |
| 28-day mortality | 1480 (11.9%) | 1049 (22.5%) | 10.5 | Descriptive; not a causal effect of TTE |
| 90-day mortality | 2095 (16.9%) | 1317 (28.2%) | 11.3 | Descriptive; not a causal effect of TTE |

**Supplementary Table S2. Model diagnostics, cohort-selection audits, missingness, and external exploratory analysis (continued)**

**C4. Selection into the final analytic cohort**

| **Characteristic** | **Level** | **Not included (n=15,100), unweighted** | **Final analytic cohort (n=1,976), unweighted** | **Absolute SMD before / after IPSW** |
| --- | --- | --- | --- | --- |
| Age, years | Median [IQR] | 64.00 [52.00, 74.00] | 62.00 [50.00, 73.00] | 0.098 / 0.016 |
| BMI, kg/m² | Median [IQR] | 27.55 [23.95, 32.10] | 28.07 [24.01, 33.15] | 0.080 / 0.004 |
| SOFA score | Median [IQR] | 3.00 [2.00, 4.00] | 4.00 [2.00, 5.00] | 0.334 / 0.026 |
| APS III | Median [IQR] | 42.00 [31.00, 57.00] | 55.00 [42.00, 73.00] | 0.580 / 0.012 |
| Lactate, mmol/L | Median [IQR] | 2.30 [1.60, 3.50] | 2.40 [1.60, 4.60] | 0.245 / 0.005 |
| Creatinine, mg/dL | Median [IQR] | 1.00 [0.80, 1.50] | 1.30 [0.90, 2.30] | 0.263 / 0.004 |
| Female sex | Yes | 6114 (40.5%) | 889 (45.0%) | 0.091 / 0.028 |
| In hospital at 72 h | Yes | 14494 (96.0%) | 1933 (97.8%) | 0.106 / 0.026 |
| Mechanical ventilation | Yes | 8646 (57.3%) | 1145 (57.9%) | 0.014 / 0.027 |
| Vasopressor use | Yes | 6125 (40.6%) | 913 (46.2%) | 0.114 / 0.051 |
| Coronary artery disease | Yes | 4220 (27.9%) | 578 (29.3%) | 0.029 / 0.081 |
| Diabetes mellitus | Yes | 3972 (26.3%) | 501 (25.4%) | 0.022 / 0.022 |
| Hypertension | Yes | 9103 (60.3%) | 1107 (56.0%) | 0.086 / 0.059 |
| Chronic kidney disease | Yes | 2145 (14.2%) | 329 (16.6%) | 0.068 / 0.005 |
| COPD | Yes | 3069 (20.3%) | 449 (22.7%) | 0.058 / 0.007 |
| BMI missing | Yes | 2224 (14.7%) | 272 (13.8%) | 0.028 / 0.003 |
| Lactate missing | Yes | 3041 (20.1%) | 239 (12.1%) | 0.220 / 0.033 |
| Creatinine missing | Yes | 39 (0.3%) | 1 (0.1%) | 0.053 / 0.001 |
| Admission type | Elective/surgical | 2757 (18.3%) | 72 (3.6%) | 0.481 / 0.027 |
| Admission type | Emergency/urgent | 10710 (70.9%) | 1664 (84.2%) | 0.323 / 0.025 |
| Admission type | Observation | 1633 (10.8%) | 240 (12.1%) | 0.042 / 0.004 |
| Admission location | Clinician referral | 4369 (28.9%) | 284 (14.4%) | 0.359 / 0.024 |
| Admission location | Emergency room | 5862 (38.8%) | 722 (36.5%) | 0.047 / 0.000 |
| Admission location | Hospital transfer | 3906 (25.9%) | 834 (42.2%) | 0.350 / 0.006 |
| Admission location | Other | 509 (3.4%) | 50 (2.5%) | 0.050 / 0.045 |
| Admission location | Self referral | 454 (3.0%) | 86 (4.4%) | 0.072 / 0.003 |
| Race | Asian | 469 (3.1%) | 51 (2.6%) | 0.032 / 0.031 |
| Race | Black | 1223 (8.1%) | 145 (7.3%) | 0.029 / 0.009 |
| Race | Hispanic/Latino | 558 (3.7%) | 69 (3.5%) | 0.011 / 0.066 |
| Race | Other | 671 (4.4%) | 88 (4.5%) | 0.000 / 0.018 |
| Race | Unknown | 2284 (15.1%) | 434 (22.0%) | 0.177 / 0.026 |
| Race | White | 9895 (65.5%) | 1189 (60.2%) | 0.111 / 0.033 |
| Insurance | Medicaid | 2279 (15.1%) | 400 (20.2%) | 0.135 / 0.006 |
| Insurance | Medicare | 7503 (49.7%) | 970 (49.1%) | 0.012 / 0.010 |
| Insurance | Other | 457 (3.0%) | 52 (2.6%) | 0.024 / 0.053 |
| Insurance | Private | 4576 (30.3%) | 519 (26.3%) | 0.090 / 0.032 |
| Insurance | Unknown | 285 (1.9%) | 35 (1.8%) | 0.009 / 0.016 |

**Supplementary Table S2. Model diagnostics, cohort-selection audits, missingness, and external exploratory analysis (continued)**

**C5. PNI-SCM interaction after selection weighting**

| **Outcome** | **Weight target and specification** | **N** | **Events** | **Weight ESS** | **Ratio of HRs (95% CI)** | **P value** |
| --- | --- | --- | --- | --- | --- | --- |
| 28-day mortality | Eligible TTE-LVEF: untrimmed stabilized IPSW | 1,976 | 506 | 1461.3 | 0.79 (0.69–0.90) | <0.001 |
| 28-day mortality | Eligible TTE-LVEF: 1st/99th percentile trimmed IPSW | 1,976 | 506 | 1561.4 | 0.79 (0.69–0.91) | <0.001 |
| 28-day mortality | Final analytic cohort: untrimmed stabilized IPSW | 1,976 | 506 | 868.3 | 0.78 (0.66–0.93) | 0.005 |
| 28-day mortality | Final analytic cohort: 1st/99th percentile trimmed IPSW | 1,976 | 506 | 1074.4 | 0.80 (0.67–0.94) | 0.007 |
| 90-day mortality | Eligible TTE-LVEF: untrimmed stabilized IPSW | 1,976 | 633 | 1461.3 | 0.83 (0.72–0.95) | 0.006 |
| 90-day mortality | Eligible TTE-LVEF: 1st/99th percentile trimmed IPSW | 1,976 | 633 | 1561.4 | 0.83 (0.73–0.95) | 0.008 |
| 90-day mortality | Final analytic cohort: untrimmed stabilized IPSW | 1,976 | 633 | 868.3 | 0.81 (0.69–0.96) | 0.014 |
| 90-day mortality | Final analytic cohort: 1st/99th percentile trimmed IPSW | 1,976 | 633 | 1074.4 | 0.83 (0.70–0.97) | 0.021 |

C1-C5 notes: The target population comprised all 17,076 patients alive at the 72-hour landmark. Subpanels C1-C3 describe echocardiographic ascertainment and comparisons by eligible pre-landmark TTE-LVEF; subpanels C4-C5 address selection into the final cohort with both eligible TTE-LVEF and calculable early PNI. Among the 15,100 patients outside the final cohort, 12,407 lacked eligible TTE-LVEF and 2,693 had eligible TTE-LVEF but incomplete PNI components. Continuous variables are median [Q1, Q3]; categorical variables are n (%). Baseline summaries and crude outcomes are unweighted. For eligible pre-landmark TTE-LVEF selection, the maximum absolute SMD was 0.454 before and 0.048 after untrimmed stabilized ATE IPSW; all 39 weighted terms were below 0.10. For final-cohort selection, the maximum absolute SMD was 0.580 before and 0.081 after stabilized ATE IPSW; no covariate term remained at or above 0.10. Selection-weighted Cox models used robust sandwich variances within each of 10 imputed datasets and Rubin-rule pooling. Crude outcome differences describe selection and are not causal effects of receiving TTE. Creatinine is reported in mg/dL in Panels C2 and C4; the main baseline tables report creatinine in μmol/L. LVEF-defined SCM was unobservable without eligible TTE. APS III, Acute Physiology Score III; ATE, average treatment effect; BMI, body mass index; COPD, chronic obstructive pulmonary disease; ESS, effective sample size; ICU, intensive care unit; IPSW, inverse-probability-of-selection weighting; LVEF, left ventricular ejection fraction; PNI, prognostic nutritional index; SCM, septic cardiomyopathy; SMD, standardized mean difference; TTE, transthoracic echocardiography.

**Supplementary Table S2. Model diagnostics, cohort-selection audits, missingness, and external exploratory analysis (continued)**

**Panel D. Missingness and multiple imputation**

**D1. Variable-specific missingness and imputation methods**

| **Variable** | **MIMIC-IV cohort, missing n/N (%)** | **External cohort, missing n/N (%)** | **Imputation method** |
| --- | --- | --- | --- |
| Body mass index | 272/1,976 (13.77) | 69/272 (25.37) | PMM where missing |
| Lactate | 239/1,976 (12.10) | 7/272 (2.57) | PMM where missing |
| White blood cell count | 5/1,976 (0.25) | 0/272 (0) | PMM in MIMIC-IV |
| SOFA score | 0/1,976 (0) | 18/272 (6.62) | PMM in external cohort |
| Blood urea nitrogen | 0/1,976 (0) | 6/272 (2.21) | PMM in external cohort |
| All other retained analytic variables | 0/1,976 (0) | 0/272 (0) | Not required |

D1 notes: Missingness was assessed after prespecified physiologic range checks and before multiple imputation. Before range checks in MIMIC-IV, missing counts were 270 for BMI, 239 for lactate, and 1 for WBC; 2 BMI values and 4 WBC values outside the prespecified ranges were subsequently set to missing. Multiple imputation by chained equations used m = 10 datasets, 20 iterations, predictive mean matching for continuous covariates, and random seed 2025. Residual missingness after imputation was 0; variables with >30% missingness were not retained in multivariable models. Albumin, lymphocyte count, derived PNI, LVEF-defined SCM, outcomes, and follow-up times were complete in the analytic cohorts and were not imputed. BMI, body mass index; BUN, blood urea nitrogen; LVEF, left ventricular ejection fraction; MIMIC-IV, Medical Information Mart for Intensive Care IV; PNI, prognostic nutritional index; PMM, predictive mean matching; SCM, septic cardiomyopathy; SOFA, Sequential Organ Failure Assessment; WBC, white blood cell count.
