# Supplementary material for: LVEF-defined septic cardiomyopathy modifies the association between the prognostic nutritional index and mortality in adults with sepsis: a 72-h landmark analysis
Source: Front Nutr. 2026 Oct 6;13:1903225. doi: 10.3389/fnut.2026.1903225 (PMC13680185; doi:10.3389/fnut.2026.1903225)
Supplement: Supplementary file 3 [file Table_3.docx]

**Supplementary Table S3. Within-cohort absolute standardized mean differences between No SCM and LVEF-defined SCM groups**

| **Category** | **Characteristic** | **MIMIC-IV absolute SMD** | **External cohort absolute SMD** |
| --- | --- | --- | --- |
| Demographics and severity | Age | 0.009 | 0.062 |
|  | Female sex | 0.223 | 0.073 |
|  | Body mass index | 0.183 | 0.284 |
|  | SOFA score | 0.008 | 0.015 |
| Cardiac and nutritional markers | LVEF | 2.919 | 4.194 |
|  | Albumin | 0.029 | 0.130 |
|  | Lymphocyte count | 0.101 | 0.049 |
|  | PNI | 0.084 | 0.076 |
| Laboratory markers | Lactate | 0.378 | 0.265 |
|  | Creatinine | 0.074 | 0.041 |
|  | White blood cell count | 0.132 | 0.111 |
|  | Platelet count | 0.076 | 0.166 |
|  | Blood urea nitrogen | 0.141 | 0.159 |
| Comorbidities | Coronary artery disease | 0.615 | 0.141 |
|  | Diabetes mellitus | 0.045 | 0.172 |
|  | Hypertension | 0.024 | 0.367 |
|  | Chronic kidney disease | 0.099 | 0.118 |
|  | Chronic obstructive pulmonary disease | 0.061 | 0.157 |
| Organ support | Mechanical ventilation | 0.246 | 0.505 |
|  | Vasopressor use | 0.250 | 0.583 |

Notes: Absolute SMDs compare the No SCM and LVEF-defined SCM groups within each cohort and are based on observed pre-imputation values. For continuous variables, the SMD was calculated as the absolute difference in means divided by the pooled standard deviation; for binary variables, the pooled binomial variance was used. SMDs are descriptive effect sizes rather than significance tests. LVEF is shown for completeness but directly defines SCM status; its large SMD is therefore expected and should not be interpreted as ordinary covariate imbalance. BMI, body mass index; CKD, chronic kidney disease; COPD, chronic obstructive pulmonary disease; LVEF, left ventricular ejection fraction; MIMIC-IV, Medical Information Mart for Intensive Care IV; PNI, prognostic nutritional index; SCM, septic cardiomyopathy; SMD, standardized mean difference; SOFA, Sequential Organ Failure Assessment.
